# Identification by Tn‐seq of *Dickeya dadantii *genes required for survival in chicory plants

**DOI:** 10.1111/mpp.12754

**Published:** 2018-11-15

**Authors:** Kévin Royet, Nicolas Parisot, Agnès Rodrigue, Erwan Gueguen, Guy Condemine

**Affiliations:** ^1^ University of Lyon Université Lyon 1, INSA de Lyon, CNRS UMR 5240 Microbiologie Adaptation et Pathogénie F‐69622 Villeurbanne France; ^2^ University of Lyon INSA‐Lyon, INRA, BF2I, UMR0203 F‐69621 Villeurbanne France

**Keywords:** *Dickeya dadantii*, glycosylation, metabolism, motility, phytopathogen, soft‐rot disease, Tn‐seq

## Abstract

The identification of the virulence factors of plant‐pathogenic bacteria has relied on the testing of individual mutants on plants, a time‐consuming process. Transposon sequencing (Tn‐seq) is a very powerful method for the identification of the genes required for bacterial growth in their host. We used this method in a soft‐rot pathogenic bacterium to identify the genes required for the multiplication of *Dickeya dadantii* in chicory. About 100 genes were identified showing decreased or increased fitness in the plant. Most had no previously attributed role in plant–bacterium interactions. Following our screening, *in planta* competition assays confirmed that the uridine monophosphate biosynthesis pathway and the purine biosynthesis pathway were essential to the survival of *D. dadantii* in the plant, as the mutants ∆*carA*, ∆*purF*, ∆*purL*, ∆*guaB* and ∆*pyrE* were unable to survive in the plant in contrast with the wild‐type (WT) bacterium. This study also demonstrated that the biosynthetic pathways of leucine, cysteine and lysine were essential for bacterial survival in the plant and that RsmC and GcpA were important in the regulation of the infection process, as the mutants ∆*rsmC* and ∆*gcpA* were hypervirulent. Finally, our study showed that *D. dadantii* flagellin was glycosylated and that this modification conferred fitness to the bacterium during plant infection. Assay by this method of the large collections of environmental pathogenic strains now available will allow an easy and rapid identification of new virulence factors.

## Introduction

*Dickeya* are broad‐host‐range phytopathogenic bacteria belonging to the *Pectobacteriaceae* family (Adeolu *et al.*, 2016) which provoke soft‐rot disease in many plant species. They are the cause of considerable losses of economically important crops, such as potato, chicory and ornamentals. Studies on and identification of the virulence factors of these bacteria have been mostly performed on the model strain *Dickeya dadantii *3937, and have focused mainly on three aspects known to be important for disease development: plant cell wall‐degrading enzymes, the type III secretion system and iron metabolism (Charkowski *et al.*, 2012). The secretion of plant cell wall‐degrading enzymes has long been identified as the main bacterial virulence factor. Many studies have focused on the identification and characterization of these secreted enzymes, mostly pectinases (Hugouvieux‐Cotte‐Pattat *et al.*, 1996), of the regulators controlling their production (*kdgR*, *pecS*, *pecT*, *hns*, *gacA*), (Condemine and Robert‐Baudouy, 1991; Lebeau *et al.*, 2008; Nasser *et al.*, 2001; Reverchon *et al.*, 1994; Surgey *et al.*, 1996), of the genes whose expression is co‐regulated with that of the secreted enzyme genes (Condemine *et al.*, 1999; Reverchon *et al.*, 2002), and of the mechanism of their secretion by the type II secretion system (Condemine *et al.*, 1992). Although of less importance for *Dickeya* virulence, the same type of approach has been used to identify type III secretion system regulators and effectors (Li *et al.*, 2015; Yang CH *et al.*, 2002; Yang S *et al.*, 2010). Moreover, the struggle for iron within the plant is strong. *Dickeya dadantii* acquires this metal through the production of two siderophores: chrysobactin and achromobactin (Franza and Expert, 1991; Franza *et al.*, 1999, 2005). Omics approaches have also been used to identify genes whose expression is induced during plant infection (Chapelle *et al.*, 2015; Okinaka *et al.*, 2002; Yang *et al.*, 2004). These studies now provide a clearer picture of the complex network of factors required for *D. dadantii* virulence (Charkowski *et al.*, 2012; Reverchon *et al.*, 2016). However, these methods may have missed some important factors not targeted by the analyses, such as the genes of metabolism constantly expressed at the same level, but nevertheless essential to the survival of the bacterium in the plant. Libraries of transposon‐induced mutants of* Pectobacterium carotovorum* and *atrosepticum*, two other soft‐rot enterobacteria, have been tested on plants to find mutants showing reduced virulence (Hinton *et al.*, 1989; Lee *et al.*, 2013; Pirhonen *et al.*, 1991). These studies identified pyrimidine, purine, leucine and serine auxotrophs and mutants defective in the production or secretion of exoenzymes and in motility. Other mutants with a more complex phenotype were not characterized at this time. Moreover, the number of tested mutants was limited by the need to test each mutant individually on the plant. This type of work has never been performed on *Dickeya* strains. To acquire a more complete view of the genes required for the virulence of *Dickeya*, we used a high‐throughput sequencing of a saturated transposon library (Tn‐seq) to screen tens of thousands of random insertion mutants of *D. dadantii* in a laboratory medium and during infection of chicory. Tn‐seq involves the creation of large transposon libraries, growth of the mutants in a control and in a selective condition, sequencing of the transposon insertion sites with next‐generation sequencing, mapping of the sequence reads to a reference genome and comparison of the number of reads in each gene in the two conditions. Tn‐seq has been used extensively to reveal the essential genes required for mouse colonization by the human pathogens *Vibrio cholerae *(Fu *et al.*, 2013), *Pseudomonas aeruginosa* (Skurnik *et al.*, 2013) and *Streptococcus pneumoniae* (van Opijnen and Camilli, 2012), plant root colonization by *Pseudomonas simiae* (Cole *et al.*, 2017) and multiplication of *Pantoea stewartii* in corn xylem (Duong *et al.*, 2018). This latter bacterium relies on the massive production of exopolysaccharides (EPSs) to block water transport and cause wilting. Thus, Tn‐seq is a very powerful method for the identification of the genes required for bacterial growth in their host. By application of this technique to screen a *D. dadantii *mutant library in chicory, we have identified the metabolic pathways and bacterial genes required by a necrotrophic bacterium for growth *in planta*. Among them, we found a cluster of genes required for flagellin glycosylation, a modification known to be important for virulence in several plant‐pathogenic bacteria.

## Results and Discussion

### Characterization of *D. dadantii *3937 *Himar1* transposon library

**Figure 1:**
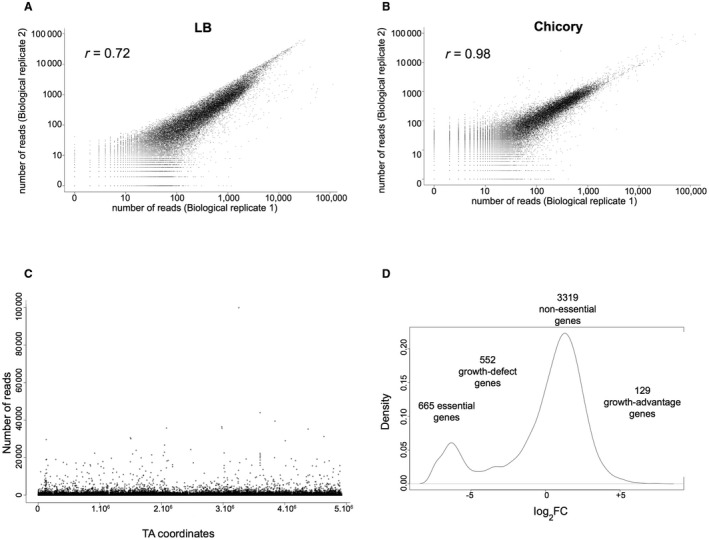
Quality control of the transposon sequencing (Tn‐seq) *Dickeya dadantii* 3937 libraries. (A, B) Biological reproducibility of the Tn‐seq results. Pairs of Tn‐seq assay results are compared, with the total number of reads per gene plotted. Analysis of DNA samples corresponding to two independent cultures of the mutant pool grown in Luria–Bertani (LB) medium (correlation coefficient *r* = 0.72) (A) and chicory (correlation coefficient *r* = 0.98) (B). Values represent average numbers of reads per gene from the pairs of biological replicates. (C) Frequency and distribution of transposon sequence reads across the entire *D. dadantii *3937 genome. The localization of transposon insertions shows no bias throughout the genome of *D. dadantii *3937. (D) Density plot of log_2_ fold change (log_2_FC; measured reads/expected reads per gene).

Many tools are available for the performance of Tn‐seq (van Opijnen and Camilli, 2013). For the Tn‐seq experiment with *D. dadantii *3937, we used a *Himar9 *mariner transposon derivative carrying *Mme*I restriction sites in the inverted repeats (IRs) and a kanamycin resistance cassette between the IRs (Wiles *et al.*, 2013). We carried out a biparental mating between *Escherichia coli* and *D. dadantii* on M63 agar medium without a carbon source and/or amino acids. We obtained approximately 300 000 colonies which were then pooled. Subsequent DNA sequencing (see below) showed the presence of transposon insertions in amino acid, vitamin, purine and pyrimidine biosynthesis pathways, demonstrating that mating on M63 minimal medium does not prevent the formation of auxotrophic mutants. To identify the essential genes, mutants were grown in Luria–Bertani (LB) medium for 10 generations. Two DNA libraries were prepared from two cultures and subjected to high‐throughput sequencing. The mariner transposon inserts into TA dinucleotides. TPP software (Dejesus *et al.*, 2015) was used to determine the number of reads at each TA site for each biological replicate. The *D. dadantii *genome has 171 791 TA sites that can be targeted by the *Himar9 *transposase. Pairs of biological replicates were compared; 37 386 and 48 119 unique insertions in TAs were detected in each sample, which corresponds to 22% and 28% density of insertion, respectively (Table 1). The mean numbers of reads over non‐zero TA sites were 406 and 268, respectively. The results were reproducible with a Pearson correlation coefficient of 72% (Fig. 1A). The location of the unique insertions showed an even distribution around the chromosome (Fig. 1C). For each gene, we calculated a log_2_ fold change (log_2_FC) corresponding to the ratio between the measured and expected number of reads. The density plot (Fig. 1D) indicates that essential (E) and non‐essential (NE) genes are easily distinguishable, confirming the good quality of our Tn‐seq libraries.

**Table 1:** Transposon sequencing (Tn‐seq) analysis of *Dickeya dadantii* 3937.

Mutant pool	Total no. of reads	No. of reads containing Tn end	No. of reads normalized*	No. of mapped reads to unique TA sites	No. of mapped reads to unique TA sites after LOESS correction	Density (%)†	Mean read count over non‐zero TA‡
LB #1	23 152 186	22 647 343	18 748 028	13 166 770 (70%)	12 904 900 (69%)	28	268
LB #2	30 105 412	27 963 154	18 748 028	15 535 291 (83%)	15 195 582 (81%)	22	406
Chicory #1	18 925 029	18 748 028	18 748 028	17 535 146 (94%)	14 906 888 (79%)	24	362
Chicory #2	27 607 717	26 555 297	18 748 028	17 477 706 (93%)	16 955 724 (90%)	23	436

* The numbers of reads containing the sequence of a Tn end were normalized for each sample according to the number of reads for the sample Chicory #1

† The *Dickeya dadantii* 3937 genome has 171 791 TA sites. The density is the percentage of TAs for which mapped reads were assigned by the TPP software.

‡ The mean value of mapped reads per TA with at least one insertion.

Next, the gene essentiality of the Tn‐seq input libraries was determined using TRANSIT software (Dejesus *et al.*, 2015). We decided to use the Hidden Markov Model (HMM) which predicts essentiality and non‐essentiality for individual insertion sites, as it has been shown to give good prediction in datasets with a density as low as 20% (Dejesus *et al.*, 2015). HMM analysis led to the identification of 665 genes essential for growth in LB medium, representing 14% of the genes of *D. dadantii *3937. Goodall *et al*. (2018) have shown that this technique overestimates the number of essential genes. Indeed, the transposon used does not allow us to distinguish between either a direct effect of the insertion or a polar effect on the downstream genes. Because some essential genes could be in an operon with non‐essential genes, some non‐essential genes could be categorized as essential. Thus, 665 must be considered as an over‐estimate of the number of essential genes. Five hundred and fifty‐two genes were categorized as growth defect genes (GD, i.e. mutations in these genes lead to a loss of fitness), 129 as growth advantage genes (GA, i.e. mutations in these genes lead to a gain of fitness) and 3319 as non‐essential genes (Fig. 1D; Table S1, see Supporting Information).

### Genes necessary for chicory leaf maceration

We used chicory leaf infection as a model to identify the *D. dadantii* genes required for growth in plant tissues. Biological duplicates were performed to ensure the reproducibility of the results. Each chicory plant was inoculated with 10^7^ bacteria from the mutant pool and, after 2 days, more than 10^10^ bacteria (representing 10 generations) were collected from the rotten tissue. Sequencing of the transposon insertion sites in these bacteria, followed by TPP analysis, indicated a density of unique insertions in TAs comparable with that of the input datasets (23%–24%). Surprisingly, the results were more highly reproducible than in LB medium, with a very high Pearson correlation coefficient of 98% (Fig. 1B). No bottleneck effect was observed as there was a strong correlation between our biological duplicates. This can be explained by the fact that 10^7^ bacteria are injected directly into the injured tissue. As we detected 37 386 and 48 119 unique insertions in TAs in LB medium, all the mutants should be present within the leaf at the beginning of the infection.

In order to test the statistical significance of the identified genes conferring a loss or a gain of fitness to *D. dadantii* *in planta*, we performed the RESAMPLING (permutation test) analysis of the TRANSIT software. The RESAMPLING method is a variation of the classical permutation test in statistics which sums the reads at all TA sites for each gene in each condition. It then calculates the difference of the sum of read counts between the input (LB medium) and output (chicory) datasets. The advantage of this statistical method is that it attributes, for each gene, an adjusted *P* value (*q*‐value). Genes with a significant difference between the total read counts in LB medium and chicory achieve a *q*‐value ≤ 0.05. The method also calculates a log_2_FC for each gene based on the ratio of the sum of read counts in the output datasets (chicory) versus the sum of read counts in the input (LB medium) datasets (Dejesus *et al.*, 2015). Applied to our Tn‐seq datasets and selecting only genes achieving a false discovery rate (FDR)‐adjusted *P* value (*q*‐value) of ≤0.05, we identified 122 genes of the 4666 required for fitness *in planta*, as shown by the volcano plot of the RESAMPLING results comparing the replicates grown in LB medium versus those *in planta* (Fig. S1, see Supporting Information). For these 122 genes, we applied an additional cut‐off by removing 20 genes with a mean read count in LB medium of less than five (less than five reads on average/TA). These 20 genes were categorized as essential or GD genes in LB medium. We also removed from the analysis six genes with a log_2_FC of between −2 and 2. By application of these criteria, we retained only 96 genes for further analysis (Table 2). Ninety‐two of these were identified as GD genes in chicory (log_2_FC ≤ 2) and the remaining four as GA genes in chicory (log_2_FC ≥ 2). A possible polar effect for genes constituting part of an operon was investigated (Table 2): if a GD gene is upstream of another GD gene in the same operon, a polar effect of insertions in the first gene on the second cannot be excluded. Some of these genes, shown in bold in Table 2, were already known to play a role in *D. dadantii* virulence, confirming the validity of the Tn‐seq approach. Using the *D. dadantii* 3937 Kyoto Encyclopedia of Genes and Genomes (KEGG) pathways database (Ogata *et al.*, 1999), we discovered that certain metabolic pathways and biological functions are very important for growth in chicory (Table S2, see Supporting Information). We highlight some of these in the following sections.

**Table 2:** Genes identified by transposon sequencing (Tn‐seq) exhibiting a growth variation from Luria–Bertani (LB) medium to chicory. Data obtained with TRANSIT software.

			HMM	RESAMPLING							
					Mean reads§						
Locus*	Gene*	Function	State in LB†	No. of TAs‡	LB	Chicory	∆Sum¶	log_2_FC**	*q*‐value††	In operon‡‡	Genes in operon (state)§§
Dda3937_00335	*glpD*	Glycerol‐3‐phosphate dehydrogenase	GD	33	650	0	−11 706	−12.56	0.00	N	
Dda3937_03379	*purL*	Phosphoribosylformyl‐glycineamide synthetase	NE	73	378	0	−21 944	−11.91	0.00	N	
**Dda3937_03564**	***opgG***	**Glucans biosynthesis protein G precursor**	**GA**	**40**	**1976**	**1**	−90 843	−11.41	**0.00**	Y	***opgG (−11.41) opgH (−9.79)***
Dda3937_00244	*purH*	Phosphoribosylaminoimidazolecarboxamide formyltransferase/IMP cyclohydrolase	NE	37	145	0	−2896	−11.25	0.00	Y	*purD (−1.66* ***) purH (−11.25)***
Dda3937_00432	*hflK*	FtsH protease regulator	GD	28	339	0	−4060	−11.12	0.03	Y	***hflK (−11.12*** *) hflC (+0.06) yjeT (−1.38)*
Dda3937_02515	*purM*	Phosphoribosylaminoimidazole synthetase	NE	21	344	0	−6188	−10.57	0.00	Y	***purM (−10.57)*** *purN (0)*
Dda3937_02627		4‐Hydroxythreonine‐4‐phosphate dehydrogenase	NE	26	129	0	−2065	−10.06	0.00	Y	***Dda3937_02627 (−10.06)*** Dda3937_02626 *(−3.77)*
Dda3937_00004	*guaB*	IMP dehydrogenase	NE	33	151	0	−3915	−9.97	0.00	N	
**Dda3937_03563**	***opgH***	**Glucans biosynthesis glucosyltransferase H**	**GA**	**62**	**1409**	**2**	**−90 073**	**−9.79**	**0.00**	Y	***opgG (−11.41) opgH (−9.79)***
Dda3937_01284	*pyrB*	Aspartate carbamoyltransferase	NE	17	159	0	−1910	−9.68	0.00	Y	***pyrB (−9.68)*** *pyrI (+1.33)*
**Dda3937_03924**	***rffG***	**dTDP‐glucose 4,6‐dehydratase**	**NE**	**23**	**317**	**1**	**−3167**	**−9.38**	**0.02**	Y	***rffG (−9.38*** *) rffH (−3.49) rfbC (−0.53) rfbD (−0.91)*
Dda3937_01389	*carB*	Carbamoyl‐phosphate synthase large subunit	NE	48	249	0	−7967	−9.23	0.00	N	
**Dda3937_03299**	***acrA***	**MexE family multidrug efflux RND transporter** **periplasmic adaptor subunit**	**NE**	**34**	**196**	**0**	**−5860**	**−9.03**	**0.00**	Y	***acrA (−9.03) acrB (−8.9)***
**Dda3937_03300**	***acrB***	**Multidrug efflux system protein**	**NE**	**89**	**422**	**1**	**−31 986**	**−8.90**	**0.00**	Y	***acrA (−9.03) acrB (−8.9)***
Dda3937_03258	*pyrE*	Orotate phosphoribosyltransferase	NE	14	175	0	−2788	−8.81	0.00	N	
Dda3937_02336	*nlpI*	Lipoprotein	GD	33	27	0	−601 000	−8.69	0.00	N	
Dda3937_02506	*nlpB (bamC)*	Outer membrane protein assembly factor BamC	NE	20	47	0	−841 000	−8.69	0.00	Y	*dapA (+2.02) * ***bamC (−8.69)***
Dda3937_04018	*pta*	Phosphate acetyltransferase	GD	36	579	2	−10 400	−8.59	0.02	N	
Dda3937_03554	*pyrC*	Dihydro‐orotase	NE	25	343	1	−7534	−8.44	0.00	N	
Dda3937_04573	*lpxM*	Acyl (myristate) transferase	NE	33	63	0	−1764	−8.31	0.00	N	
Dda3937_01116	*glnG*	Nitrogen regulation protein NR(I), two‐component system	NE	26	39	0	−629 000	−8.22	0.00	Y	***glnL (−0.2) glnG (−8.22)***
Dda3937_02099	*purF*	Amidophosphoribosyltransferase	NE	32	107	0	−2779	−8.19	0.00	Y	***purF*** ***(−8.19)*** *cvpA (−1.92)*
Dda3937_04019	*ackA*	Acetate kinase A and propionate kinase 2	NE	29	45	0	−1063	−8.16	0.00	Y	*Dda3937_04020 (−2.48) * ***ackA (−8.16)***
Dda3937_02189	*yejM*	Membrane‐anchored periplasmic protein, alkaline phosphatase superfamily	GA	34	4160	15	−99 478	−8.08	0.00	Y	*yejL (0) * ***yejM (−8.08)***
Dda3937_01390	*carA*	Carbamoyl‐phosphate synthase small subunit	NE	21	69	0	−956 000	−8.05	0.00	N	
Dda3937_01426	*ptsI*	Phosphoenolpyruvate‐protein phosphotransferase of PTS system	NE	33	45	0	−1176	−7.85	0.00	Y	*crr (−2.66) * ***ptsI (−7.85)*** *ptsH (0)*
Dda3937_00161	*cysQ*	3'(2'),5'‐Bisphosphate nucleotidase	NE	16	44	0	−434 000	−7.81	0.02	N	
Dda3937_00210	*cysI*	Sulfite reductase β subunit	NE	40	252	1	−7515	−7.65	0.00	Y	***cysH (−8.93) cysI (−7.65) cysJ (−6.25)***
Dda3937_04075	*lysR*	LysR family transcriptional regulator	NE	13	2385	13	−18 976	−7.51	0.00	N	
Dda3937_02526	*yidR*	Conserved protein	NE	18	50	0	−591 000	−7.50	0.00	N	
Dda3937_03888	*metB*	Cystathionine γ‐synthase	NE	21	118	1	−1881	−7.34	0.01	Y	***metB (−7.34)*** *metL (−3.23)*
Dda3937_00195	*relA*	(p)ppGpp synthetase I/GTP pyrophosphokinase	NE	55	256	2	−11 683	−7.12	0.00	Y	***relA (−7.12)*** *rumA (−1.33)*
**Dda3937_02532**	***lfcR***	**Fructose repressor FruR, LacI family**	**NE**	**15**	**399**	**3**	**−4756**	**−7.04**	**0.00**	N	
Dda3937_02226	*fliF*	Flagellar M‐ring protein fliF	NE	46	476	4	−18 898	−7.02	0.00	Y	***fliF (−7.02*** *) fliG (−4.26) fliH (−3.92* ***) fliI (−6.56) fliJ (−5.44)*** * fliK (−4.71)*
Dda3937_02206	*flgE*	Flagellar hook protein flgE	NE	50	597	5	−29 608	−7.00	0.00	Y	***flgE (−7)*** ***flgF (−4.76) flgG (−5.91)***
Dda3937_04507	*gnd*	Phosphogluconate dehydrogenase (NADP(+)‐dependent, decarboxylating)	GD	36	7	0	−190 000	−6.91	0.00	N	
Dda3937_00697	*degQ*	Protease	NE	28	80	1	−956 000	−6.87	0.01	N	
Dda3937_03631	*trxB*	Thioredoxin‐disulfide reductase	GD	25	16	0	−257 000	−6.85	0.03	N	
Dda3937_00361	*yrfF (igaA)*	Intracellular growth attenuator protein	GD	38	22	0	−430 000	−6.78	0.03	N	
Dda3937_00588	*cysB*	Transcriptional dual regulator, *O*‐acetyl‐l‐serine‐binding protein	NE	29	90	1	−2504	−6.75	0.00	N	
Dda3937_03783	*prc*	Carboxy‐terminal protease for penicillin‐binding protein 3	NE	46	243	2	−11 557	−6.71	0.00	Y	***prc (−6.71)*** *proQ (−1.82)*
Dda3937_00433	*hflX*	Predicted GTPase	GD	27	16	0	−187 000	−6.69	0.04	N	
Dda3937_03427	*fliC*	Flagellar filament structural protein (flagellin)	NE	33	96	1	−1520	−6.61	0.03	Y	
Dda3937_02223	*fliI*	Flagellum‐specific ATP synthase fliI	NE	42	236	3	−7009	−6.56	0.00	Y	***fliF (−7.02*** *) fliG (−4.26) fliH (−3.92* ***) fliI (−6.56) fliJ (−5.44)*** * fliK (−4.71)*
Dda3937_04419	*hdfR*	DNA‐binding transcriptional regulator	NE	29	117	1	−3241	−6.34	0.00	N	
Dda3937_00209	*cysJ*	Sulfite reductase α subunit	NE	41	180	2	−6746	−6.25	0.00	Y	***cysH (−8.93) cysI (−7.65) cysJ (−6.25)***
Dda3937_02209	*flgH*	Flagellar L‐ring protein flgH	NE	23	586	8	−13 875	−6.22	0.01	Y	***flgH (−6.22) flgI (−5.49)*** * flgJ (−7.16)*
Dda3937_02246	*fabF*	β‐Ketoacyl‐[acyl‐carrier‐protein] synthase II	GD	41	10	0	−273 000	−6.15	0.00	N	
Dda3937_00301	*uvrD*	ATP‐dependent DNA helicase UvrD/PcrA	NE	42	29	0	−678 000	−6.11	0.00	N	
Dda3937_02212	*flgK*	Flagellar hook‐associated protein flgK	NE	63	116	2	−4808	−6.07	0.00	Y	***flgK (−6.07)*** *flgL (−5.58)*
Dda3937_04046	*purU*	Formyltetrahydrofolate deformylase	NE	28	51	1	−1105	−5.84	0.00	N	
Dda3937_03965	*flhA*	Predicted flagellar export pore protein	NE	49	106	2	−3532	−5.80	0.00	Y	*flhE (−0.89* ***) flhA (−5.8) flhB (−5.31)*** *Dda3937_04633 (−1) cheZ (−3.29) cheY (−4.52) * ***cheB (−5.14) cheR (−4.67)***
Dda3937_02205	*flgD*	Flagellar basal‐body rod modification protein flgD	NE	22	227	4	−4905	−5.73	0.01	Y	*flgB (−3.45) flgC (−6.38) * ***flgD (−5.73)***
Dda3937_01352	*leuC*	3‐Isopropylmalate dehydratase large subunit	NE	21	139	3	−2457	−5.73	0.01	Y	***leuA (−4.69*** *) * *** leuB (−4.63) leuC (−5.73*** *) leuD (−6.26)*
Dda3937_02784	*flhC*	Flagellar transcriptional activator flhC	NE	20	477	9	−11 222	−5.66	0.01	Y	***flhC (−5.66)*** *flhD (−4.1)*
Dda3937_02782	*motB*	Flagellar motor rotation protein motB	NE	40	109	2	−4067	−5.55	0.01	Y	***motA (−5.06) motB (−5.55) cheA (−4.89)*** *cheW (−5.39)*
Dda3937_02210	*flgI*	Flagellar P‐ring protein flgI	NE	26	163	4	−3191	−5.49	0.00	Y	***flgH (−6.22) flgI (−5.49) *** *flgJ (−7.16)*
Dda3937_02222	*fliJ*	Flagellar protein fliJ	NE	14	182	4	−2486	−5.44	0.03	Y	***fliF (−7.02*** *) fliG (−4.26) fliH (−3.92* ***) fliI (−6.56) fliJ (−5.44)*** * fliK (−4.71)*
Dda3937_02219	*fliM*	Flagellar motor switch protein fliM	NE	27	143	3	−3339	−5.40	0.00	Y	*fliL (−4.17)* ***fliM (−5.4)*** *fliN (−4.78) fliO (−6.89) fliP (−4.78) fliQ (−3.12) * ***fliR (−4.56)***
Dda3937_02774	*flhB*	Flagellar biosynthesis protein flhB	NE	32	186	5	−4712	−5.31	0.00	Y	*flhE (−0.89* ***) flhA (−5.8) flhB (−5.31)*** *Dda3937_04633 (−1) cheZ (−3.29) cheY (−4.52) * ***cheB (−5.14) cheR (−4.67)***
Dda3937_02777	*cheB*	Chemotaxis response regulator protein‐glutamate methylesterase CheB	NE	31	282	8	−7682	−5.14	0.00	Y	*flhE (−0.89* ***) flhA (−5.8) flhB (−5.31)*** *Dda3937_04633 (−1) cheZ (−3.29) cheY (−4.52) * ***cheB (−5.14) cheR (−4.67)***
Dda3937_02783	*motA*	Flagellar motor rotation protein motA	NE	24	39	1	−834 000	−5.06	0.00	Y	***motA (−5.06) motB (−5.55) cheA (−4.89)*** *cheW (−5.39)*
Dda3937_00565	*tonB*	TonB protein	NE	14	106	3	−2062	−5.00	0.05	N	
Dda3937_00427	*fbp*	Fructose‐bisphosphatase	GA	33	805	27	−28 026	−4.92	0.01	N	
Dda3937_02781	*cheA*	Chemotaxis protein CheA	NE	50	151	5	−5838	−4.89	0.00	Y	***motA (−5.06) motB (−5.55) cheA (−4.89)*** *cheW (−5.39)*
Dda3937_03422		Carbamoyl‐phosphate synthase small subunit	NE	43	379	13	−11 713	−4.85	0.02	Y	*Dda3937_03422 (−4.85) Dda3937_03421 (−0.71)*
Dda3937_02577	*lysA*	Diaminopimelate decarboxylase	NE	23	332	0	−3989	−4.79	0.00	N	
Dda3937_02207	*flgF*	Flagellar basal‐body rod protein flgF	NE	21	35	1	−671 000	−4.76	0.00	Y	***flgE (−7)*** ***flgF (−4.76) flgG (−5.91)***
Dda3937_02230	*fliD*	Flagellar hook‐associated protein fliD	NE	47	93	3	−2506	−4.75	0.00	N	
Dda3937_04301	*leuA*	2‐Isopropylmalate synthase	NE	36	35	1	−944 000	−4.69	0.02	Y	***leuA (−4.69*** *) * ***leuB (−4.63) leuC (−5.73*** *) leuD (−6.26)*
Dda3937_02778	*cheR*	Chemotaxis protein methyltransferase CheR	NE	30	462	18	−8882	−4.67	0.05	Y	*flhE (−0.89* ***) flhA (−5.8) flhB (−5.31)*** *Dda3937_04633 (−1) cheZ (−3.29) cheY (−4.52) * ***cheB (−5.14) cheR (−4.67)***
Dda3937_02228	*fliT*	Flagellar biosynthesis protein fliT	GD	16	8	0	−95 000	−4.63	0.05	Y	*fliS (−6.36) * ***fliT (−4.63)***
Dda3937_04404	*leuB*	3‐Isopropylmalate dehydrogenase	NE	16	285	12	−3835	−4.63	0.05	Y	***leuA (−4.69*** *) * ***leuB (−4.63) leuC (−5.73*** *) leuD (−6.26)*
Dda3937_02214	*fliR*	Flagellar biosynthesis protein fliR	NE	33	268	11	−5653	−4.56	0.00	Y	*fliL (−4.17)* ***fliM (−5.4)*** *fliN (−4.78) fliO (−6.89) fliP (−4.78) fliQ (−3.12) * ***fliR (−4.56)***
**Dda3937_03727**	***kduI***	**4‐Deoxy‐l‐threo‐5‐hexosulose‐uronate ketol‐isomerase**	**NE**	**26**	**70**	**3**	**−2015**	**−4.54**	**0.03**	N	
Dda3937_03267		*O*‐Antigen, teichoic acid lipoteichoic acids export membrane protein	ES	107	89	4	−1181	−4.33	0.05	Y	***Dda3937_03267 (−4.33)*** *Dda3937_03268 (−1.07)*
Dda3937_00415	*epd*	d‐Erythrose 4‐phosphate dehydrogenase	NE	26	316	16	−4793	−4.27	0.02	N	
Dda3937_02337	*pnp*	Polynucleotide phosphorylase/polyadenylase	GD	50	5	0	−105 000	−3.97	0.00	N	
Dda3937_01683	*purK*	N5‐Carboxyaminoimidazole ribonucleotide synthase	NE	16	90	0	−722 000	−3.49	0.01	Y	*purE (−5.75) * ***purK (−3.49)***
Dda3937_00689	*yrbF (mlaF)*	Predicted toluene transporter subunit	GA	9	1254	114	−15 962	−3.47	0.01	Y	***yrbF (−3.47)*** *yrbE (−1.48) yrbD (−3.09) * ***yrbC (−2.81)*** * yrbB (−0.24))*
Dda3937_02829	*helD*	DNA helicase IV	NE	26	99	9	−1803	−3.46	0.01	N	
Dda3937_02252	*ptsG*	PTS system glucose‐specific IICB component	NE	37	81	8	−2928	−3.38	0.03	N	
**Dda3937_00726**	***tolC***	**Transport channel**	**NE**	**34**	**184**	**0**	**−3304**	**−3.35**	**0.00**	N	
Dda3937_02363	*clpA*	ATP‐dependent Clp protease ATP‐binding subunit	NE	44	64	8	−1793	−3.02	0.03	Y	*clpS (−2.07* ***) clpA (−3.02)***
Dda3937_02470	*corC*	Magnesium and cobalt ions transport	NE	13	159	21	−1377	−2.90	0.02	Y	*lnt (+3.02)* ***corC (−2.09)***
Dda3937_00692	*yrbC (mlaC)*	Predicted ABC‐type organic solvent transporter	GA	23	740	106	−16 493	−2.81	0.01	Y	***yrbF (−3.47)*** *yrbE (−1.48) yrbD (−3.09) * ***yrbC (−2.81)*** * yrbB (−0.24)*
Dda3937_02045	*envC*	Murein hydrolase activator	NE	17	71	12	−825 000	−2.59	0.00	N	
Dda3937_01807	*nuoM*	NADH‐quinone oxidoreductase subunit M	NE	29	57	10	−1130	−2.47	0.03	Y	*nuoN (−2.01* ***) nuoM (−2.47)***
Dda3937_03668	*sufB*	Fe‐S cluster assembly protein	NE	32	116	21	−3581	−2.44	0.00	Y	***sufB (−2.44*** *) sufA (−1.47)*
Dda3937_02080	*trkH*	Potassium uptake protein	NE	36	65	13	−1047	−2.33	0.05	Y	*pepQ (−0.21) yigZ (+0.1) trkH (−2.33) hemG (+1.15)*
**Dda3937_03042**	***fct***	**Ferrichrysobactin outer membrane receptor**	**NE**	**80**	**244**	**51**	**−14 622**	**−2.25**	**0.01**	N	
Dda3937_01287	*argI*	Ornithine carbamoyltransferase	NE	24	279	59	−4383	−2.23	0.03	N	
Dda3937_02456	*rsmC*	Global regulatory protein RsmC	NE	10	116	221,705	2 659 067	10.90	0.028	N	
Dda3937_03858	*gcpA*	Hypothetical protein	GA	55	3728	140,136	9 002 975	5.23	0.00	N	
Dda3937_03971	*mltD*	Outer membrane‐bound lytic murein transglycosylase D	NE	46	276	10,885	445 590	5.30	0.00	N	
Dda3937_00363	*mrcA*	Penicillin‐binding protein 1A (PBP1A)	NE	53	85	468	16 879	2.47	0.021	N	

* Genes for which a role in *D. dadantii* virulence has been described before are in bold. Underlined genes have been deleted to study the mutants in further analysis.

† State of each gene in LB defined by the TRANSIT software using an Hidden Markov Model: NE, Non‐Essential; GD, Growth Defect; E, Essential; GA, Growth Advantage.

‡ Number of TAs in the gene.

§ Mean reads per TA site for a gene in each growth condition.

¶ Difference of reads between chicory and LB growth condition.

** Ratio of reads between chicory and LB condition expressed in log_2_.

†† *P*‐values adjusted for multiple comparisons using the Benjamini‐Hochberg procedure (See Transit manual).

‡‡ Presence of the gene in an operon (Yes or No).

§§ Operon structure determined by analysis of *D. dadantii* 3937 RNA‐seq datasets from Jiang X *et al*, Environ Microbiol. 2016 Nov;18(11):3651‐3672. log_2_FC for each gene in operon are indicated in brackets, genes considered to be essential in chicory are indicated in bold (*q*‐value < 0.05).

### Analysis of the genes of *D. dadantii* required for plant colonization

#### (i) *Metabolism*

**Figure 2:**
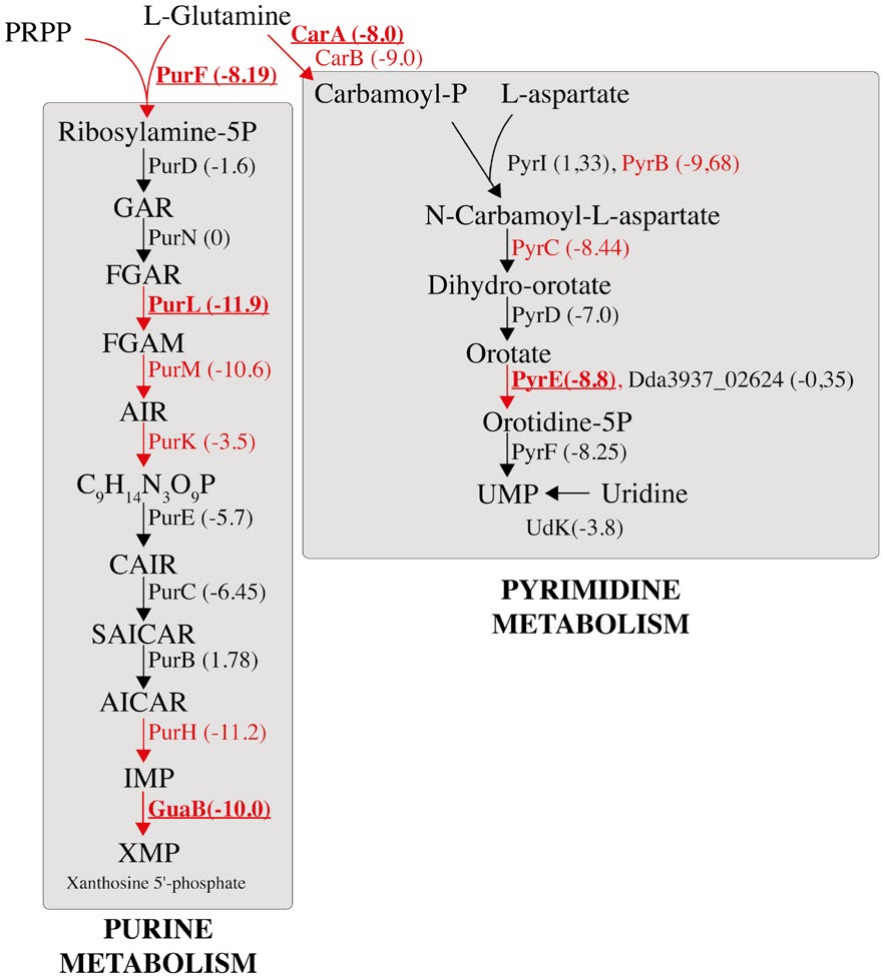
Scheme of the purine and pyrimidine biosynthesis pathways in *Dickeya dadantii* which produce XMP (purine metabolism) and UMP (pyrimidine metabolism) from l‐glutamine. Pathways are drawn based on the *D. dadantii* 3937 Kyoto Encyclopedia of Genes and Genomes (KEGG) database. The growth defect (GD) genes in chicory that pass the permutation test (*q*‐value ≤ 0.05) are indicated in red. The genes for which the GD phenotype was tested and confirmed with in‐frame deletion mutants are shown in bold. The log_2_ fold change (log_2_FC) of read numbers between chicory and Luria–Bertani (LB) medium for each gene is indicated in parentheses. Some genes do not pass the permutation test (in black), but have a strongly negative log_2_FC. PRPP, 5‐phosphoribosyl‐1‐pyrophosphate; GAR, 5′‐phosphoribosyl‐1‐glycinamide; FGAR, 5′‐phosphoribosylformylglycinamide; FGAM, 5′‐phosphoribosyl‐*N*‐formylglycinamide; AIR, 5′‐phosphoribosyl‐5‐aminoimidazole; CAIR, 5′‐phosphoribosyl‐5‐aminoimidazole carboxylic acid; SAICAR, 5′‐phosphoribosyl‐4‐(*N*‐succino‐carboxamide)‐5‐aminoimidazole; AICAR, 5‐aminoimidazole‐4‐carboxamide ribonucleotide; IMP, inosine monophosphate; XMP, xanthine monophosphate; UMP, uridine monophosphate. [Colour figure can be viewed at wileyonlinelibrary.com]

Chicory plants appear to provide conditions in which amino acids, nucleic acids and some vitamins (pyridoxal) are scarce. Of the 92 genes identified as GD genes *in planta*, eight are involved in purine and seven in pyrimidine metabolism (Table S2). In the purine metabolism pathway, the inosine monophosphate (IMP) biosynthesis pathway, which produces IMP from l‐glutamine and 5‐phosphoribosyl diphosphate, is particularly important for *D. dadantii* *in planta*, as five of the 10 genes of this pathway are significant GD genes *in planta *(Fig. 2). IMP is the precursor of adenine and guanine, and IMP can be converted into xanthosine 5′‐phosphate (XMP) by the IMP dehydrogenase GuaB. The *guaB *gene is also a GD gene *in planta*, with a strong log_2_FC of –10.06 (Fig. 2). In pyrimidine synthesis, the uridine monophosphate (UMP) biosynthesis pathway, which converts l‐glutamine to UMP, a precursor of uracyl, is very important *in planta*, as *carAB*, *pyrB*, *pyrC* and *pyrE*, involved in this enzymatic pathway, are all required for growth *in planta* (Fig. 2). This pyrimidine biosynthesis pathway is specific to bacteria. It is noteworthy that, in the human pathogen *S. pneumoniae*, mutants of this pathway have a fitness defect in the nasopharynx of infected mice (van Opijnen and Camilli, 2012). Hence, it seems that the pyrimidine biosynthesis pathway is particularly important for the multiplication of some bacterial species in the host.

Mutants in genes involved in the synthesis of sulfur‐containing amino acids (*cysIJQ*, *metB*), lysine (*lysA*) and leucine (*leuABC*) are disadvantaged in chicory (Table 2; Fig. S2, see Supporting Information). These amino acids are known to be present in low concentrations in plant tissues (Azevedo *et al.*, 1997). Other amino acids seem to be present in sufficient quantities for the growth of *D. dadantii *auxotrophs. A low level of certain amino acids probably induces a stringent response in the bacterium. Reduced growth in the plant of the *relA* mutant, unable to synthesize the alarmone ppGpp, supports this hypothesis. Glucose is one of the main sugars in plant tissue, present as a circulating sugar or a cellulose degradation product (Buysse and Merckx, 1993). Mutants in the PTS glucose transport system genes *ptsI* and *ptsG* showed a reduced growth in the bacterium (Table 2), indicating their importance *in planta*.

The degradation of cell wall pectin by a battery of extracellular enzymes is the main determinant of *Dickeya* pathogenicity. Mutants unable to produce or to secrete these enzymes by the type II secretion system were not disadvantaged in chicory, as these mutants could use for their growth the pectin degradation compounds produced by enzymes secreted by other bacteria. The redundancy of oligogalacturonate‐specific porins (KdgM and KdgN) and inner membrane transporters (TogT and TogMNABC) allows the entry of these compounds into the bacterium, even in a mutant in one of these transport systems. However, *kduI* mutants, blocked in the intracellular part of the pectin degradation pathway, show limited growth *in planta*, confirming the importance of the pectin degradation pathway in disease progression.

#### (ii) *Stress resistance*

Plants are a hostile environment for bacteria having to cope with antimicrobial peptides, reactive oxygen species (ROS), toxic compounds and acidic pH (Reverchon and Nasser, 2013). We observed that the pump AcrABTolC, which can efflux a wide range of compounds (Ravirala *et al.*, 2007), is important for survival in chicory (Fig. S2). Stress can lead to the accumulation of phospholipids in the outer membrane. This accumulation makes the bacterium more sensitive to small toxic molecules (Malinverni and Silhavy, 2009). This phospholipid accumulation probably occurs when the bacterium infects chicory, as *mlaC* and *mlaF* mutants, which are unable to transport phospholipid from the outer to the inner membrane, show a reduced growth *in planta*. The production of EPSs has been shown to protect bacteria during the first steps of infection (Condemine *et al.*, 1999). We observed that *rffG* and *wzx* mutants, unable to synthesize EPS, show a growth defect in chicory. A set of genes required to repair or degrade altered proteins (*clpA*, *degQ*, *trxB*) is also important for survival *in planta*. No gene directly involved in the detoxification of ROS was detected in our analysis. However, ROS can create DNA damage. The two helicases involved in DNA repair, UvrD and HelD, give a growth advantage *in planta*. Osmoregulated periplasmic glycans (OPGs) are polymers of glucose found in the periplasm of α‐, β‐ and γ‐proteobacteria. Their exact role is unknown, but their absence leads to avirulence in certain bacteria, such as *D. dadantii* (Page *et al.*, 2001). This absence induces a membrane stress that is sensed and transduced by the Rcs envelope stress response system. This system controls the expression of many genes, including those involved in motility and those encoding plant cell wall‐degrading enzymes through the RsmA‐RsmB system (Bouchart *et al.*, 2010; Madec *et al.*, 2014; Wu *et al.*, 2014). Thus, mutants defective in OPG synthesis are expected to show reduced virulence. Indeed, in our experiment, mutants in the two genes involved in OPG synthesis, *opgG* and *opgH*, were non‐competitive in chicory (Table 2).

#### (iii) *Iron uptake*

*Dickeya dadantii* produces two types of siderophore, achromobactin and chrysobactin, which are required for the development of maceration symptoms in the iron‐limited environment of plant hosts (Franza and Expert, 2013). Once the iron is loaded, the siderophores are imported into the bacterium. Import through the outer membrane requires a specific outer membrane channel and the energy‐transducing complex formed by TonB, ExbB and ExbD. Although the absence of synthesis of one of the siderophores can be compensated for by the presence of siderophores secreted by other bacteria in the growth medium, mutants of the TonB complex are totally unable to acquire iron and thus are unable to grow in the plant. Consequently, *tonB* is essential in chicory, whereas the genes coding for siderophore synthesis or secretion are not. Similarly a mutant devoid of the iron‐loaded chrysobactin transport gene (*fct*) is non‐competitive.

#### (iv) *Regulation*

Mutants in several genes controlling virulence factor production show a growth defect in the plant. The master regulator FlhDC acts as a regulator of both flagella and virulence factor synthesis in many bacteria, such as *Yersinia ruckeri*, *Edwardsiella tarda* and *Ralstonia solanacearum *(Jozwick *et al.*, 2016; Tans‐Kersten *et al.*, 2004; Xu *et al.*, 2014). In *D. dadantii*, FlhDC has recently been shown to control, in addition to flagellar motility, a type III secretion system and virulence factor synthesis through several pathways (Yuan *et al.*, 2015). We observed that *flhC* gives a certain growth advantage in chicory. In addition, we discovered that some genes regulating *flhDC* in other bacteria regulate *D. dadantii* virulence, probably by controlling *flhDC* expression. *rsmC* is a poorly characterized gene in *D. dadantii*, but has been studied in *P. carotovorum*. It negatively controls motility and extracellular enzyme production through the modulation of the transcriptional activity of FlhCD (Chatterjee *et al.*, 2009). HdfR is a poorly characterized LysR family regulator that controls the *std* fimbrial operon in *S. enterica* and FlhDC expression in *E. coli* (Ko and Park, 2000). *rsmC* mutants were over‐represented in chicory (Fig. S2), indicating an increase in virulence for these mutants. *hdfR* conferred fitness benefits during growth in chicory and could also act in *D. dadantii* as an activator of *flhDC *expression.

The GGDEF proteins are cyclic diguanosine monophosphate (c‐di‐GMP) synthases and their genes are often located next to their cognate EAL diguanylate phosphodiesterase gene. *ecpC* (*yhjH*) encodes an EAL protein which has been shown to activate virulence factor production in *D. dadantii* (Yi *et al.*, 2010). *gcpA*, which is located next to *ecpC*, encodes a GGDEF protein. The role of *gcpA* in *D. dadantii* virulence has been described recently (Yuan *et al.*, 2018). We observed that *gcpA* mutants (Dda_03858) were over‐represented in chicory (Table 2). This increased virulence, with an opposite phenotype to that described for the *ecpC* mutants, indicates that the overproduction of c‐di‐GMP could reduce *D. dadantii *virulence.

Of the 18 regulators of the LacI family present in *D. dadantii*, four were found to be involved in plant infection (Van Gijsegem *et al.*, 2008). One of these, LfcR, which has been found to play a major role in the infection of chicory, Saintpaulia and *Arabidopsis*, was seen to be important for chicory infection in our experiment. LfcR is a repressor of adjacent genes (Van Gijsegem *et al.*, 2008). Surprisingly none of these genes appeared to play a role in chicory infection, suggesting that there are other targets of LfcR that remain to be discovered.

Finally, it is worth mentioning that the *ackA* and *pta* genes are GD genes *in planta*. These genes constitute the reversible Pta‐AckA pathway. The steady‐state concentration of acetyl‐phosphate (acetyl‐P), a signalling molecule in bacteria, depends on the rate of its formation catalysed by Pta and of its degradation catalysed by AckA (Wolfe, 2005). The GD phenotype of *D. dadantii* *ackA *and *pta* mutants during infection suggests that acetyl‐P might play a crucial signalling role in the adaptation of *D. dadantii* to plant tissue.

#### (v) *Motility*

Motility is an essential virulence factor of *D. dadantii* necessary for the bacterium to move across the surface of the leaf, to enter wounds and to propagate within plant tissue (Antunez‐Lamas *et al.*, 2009; Jahn *et al.*, 2008; Rio‐Alvarez *et al.*, 2015). Accordingly, all the genes required for flagella synthesis, the flagella motor and the genes regulating their synthesis (*flhC*, *flhD*, *fliA*) (see above) are necessary for fitness during chicory infection (Fig. S2). All the genes responsible for the transduction of the chemotaxis signal (*cheA*, *cheB*, *cheR*, *cheW*, *cheX*, *cheY* and *cheZ*) also confer benefits *in planta* (Table 2). No methyl‐accepting chemoreceptor gene mutant was found. Like other environmental bacteria, *D. dadantii* encodes many such proteins. They probably have a certain redundancy in the recognized signal which prevented their detection in our screen.

### *Dickeya dadantii* flagellin is modified by glycosylation

**Figure 3:**
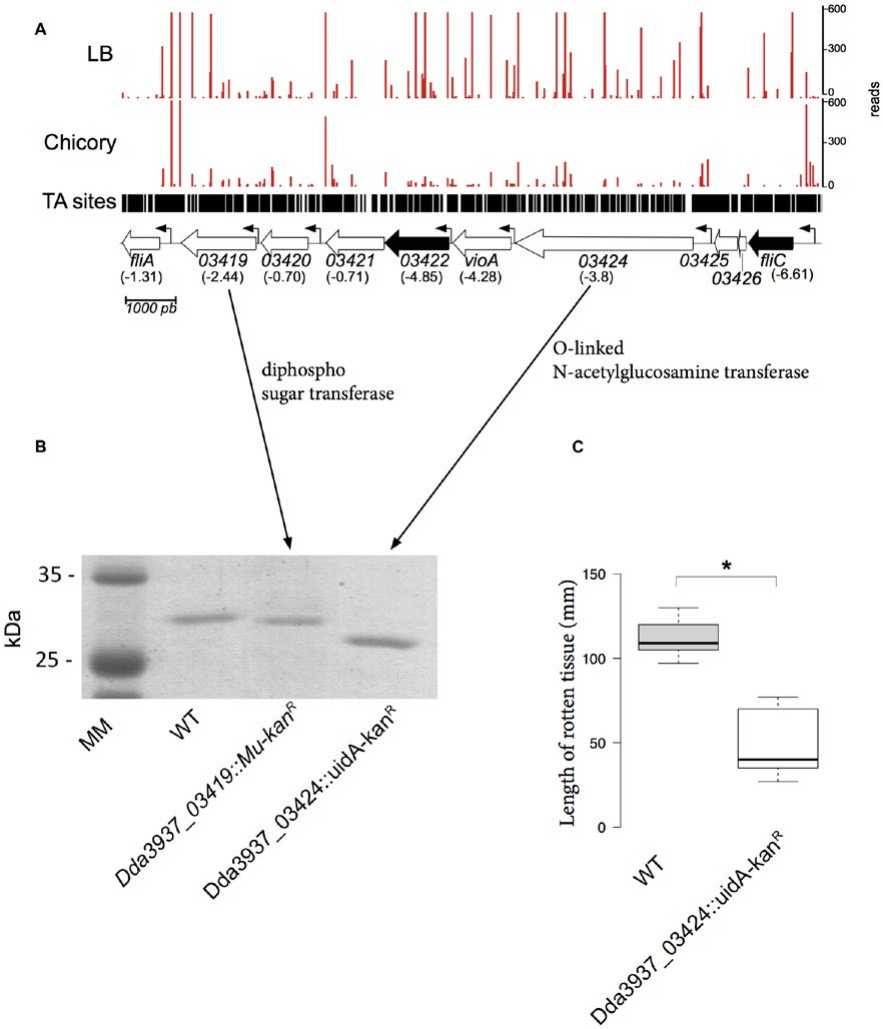
Modification of FliC revealed by transposon sequencing (Tn‐seq) analysis and sodium dodecylsulfate‐polyacrylamide gel electrophoresis (SDS‐PAGE). (A) The importance of six genes located between *fliA *and *fliC* for growth in chicory; log_2_ fold changes (log_2_FC) are indicated in parentheses. Dda3937_03425 and Dda3937_03426 are duplicated transposase genes that have been removed from the analysis. Black arrow, growth defect (GD) in chicory (*q*‐value ≤ 0.05); white arrow, genes that do not pass the permutation test (*q*‐value > 0.05). Small arrows indicate the presence of a promoter. (B) Analysis by SDS‐PAGE of FliC produced by the wild‐type (WT, lane 2), A3422 (lane 3) and A4277 (lane 4) strains. (C) Maceration of celery leaves by the WT and A4277 (glycosylation) mutant. The length of rotten tissue was measured at 48 h post‐infection. Boxplots were generated by BoxPlotR from nine data points. The calculated median value is 109 for the WT strain and 40 for the A4277 strain. Centre lines show the medians; box limits indicate the 25th and 75th percentiles as determined by R software; whiskers extend 1.5 times the interquartile range from the 25th and 75th percentiles. *indicates a significant difference relative to the WT (*P* < 0.05). Statistical analysis was performed with the Mann–Whitney *U*‐test. [Colour figure can be viewed at wileyonlinelibrary.com]

A group of six genes located between *fliA* and *fliC* retained our interest, as insertions in one of these genes led to a growth defect in chicory (Fig. 3A). This effect does not result from insertions in the first gene of the group as they are not expressed in an operon (Jiang *et al.*, 2016). Dda3937_03424 encodes an *O*‐linked *N*‐acetylglucosamine transferase and Dda3937_03419 encodes a protein with a nucleotide diphospho sugar transferase predicted activity. The others could be involved in the modification of sugars (predicted function of: Dda3937_03423, nucleotide sugar transaminase; Dda3937_03422, carbamoyl phosphate synthase; Dda3937_03421, oxidoreductase; Dda3937_03420, methyltransferase). Their location led us to suppose that this group of genes could be involved in flagellin glycosylation. Analysis by sodium dodecylsulfate‐polyacrylamide gel electrophoresis (SDS‐PAGE) of FliC produced by the wild‐type (WT) and mutants in the two glycosyltransferase genes (Dda3937_03424 and Dda3937_03419) revealed that, in the last two strains, the molecular weight of the protein diminished (Fig. 3B). The molecular weight determined by mass spectroscopy was 28 890 Da for FliC_A4277_, 31 034 Da for FliC_A3422_ and 32170 Da for WT FliC. Thus, the presence in the gene cluster of two glycosyltransferases suggests that, in the WT strain, FliC is modified by multiple glycosylation with a disaccharide. The absence of any modification did not affect *D. dadantii* motility (data not shown). The flagellin of the plant pathogens *Pseudomonas syringae* pv *tabaci* and *Burkholderia cenocepacia* are also glycosylated, and the absence of this modification lowered the ability of these bacteria to cause disease on tobacco and *Arabidopsis*, respectively (Khodai‐Kalaki *et al.*, 2015; Taguchi *et al.*, 2010). Accordingly, in *D. dadantii*, FliC modification appears to be important for the multiplication of the bacterium in the plant (Fig. 3C).

### Validation of the Tn‐seq results

**Figure 4:**
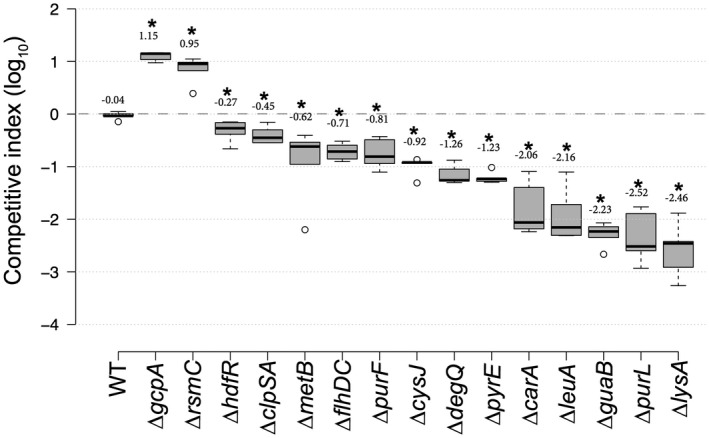
Competitive index (CI) of several mutant strains. CI values were determined in chicory leaves as described in Experimental details. Each value is the mean of five experiments. Centre lines show the medians; box limits indicate the 25th and 75th percentiles as determined by R software; whiskers extend 1.5 times the interquartile range from the 25th and 75th percentiles; outliers are represented by dots. *n* = 5 sample points. Numbers above the boxes indicate the average CI in log_10_. *Significant difference relative to the wild‐type (WT) (*P* < 0.05). Statistical analysis was performed with the Mann–Whitney *U*‐test.

To validate the Tn‐seq results, we performed co‐inoculation experiments in chicory leaves with the WT strain and various mutants in GA genes (*gcpA *and* rsmC*) or GD genes (*hdfR*, *clpSA*, *metB*, *flhDC*, *purF*, *cysJ*, *degQ*, *pyrE*, *carA*, *leuA*, *guaB*, *purl* and *lysA*) in a 1 : 1 ratio. We calculated a competitive index (CI) by counting the numbers of each type of bacteria in rotten tissue after 24 h. We confirmed the ability of ∆*rsmC* and ∆*gcpA* to overgrow the WT strain. However, the WT strain overgrew the other in‐frame deletion mutants that were tested (Fig. 4). The lowest CIs were observed with the mutants in biosynthetic pathways, such as ∆*leuA*, ∆*guaB*, ∆*purL* and ∆*lysA.*

**Figure 5:**
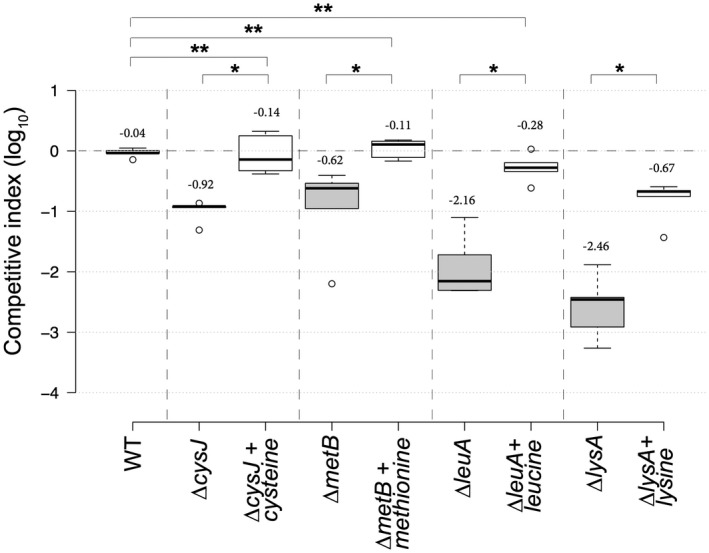
Complementation of auxotrophic mutants *in planta*. Each leaf was inoculated with 10^6^ bacteria. The length of rotten tissue was measured after 24 h. Bacteria were injected into the wounded leaf with or without amino acid. Centre lines show the medians; box limits indicate the 25th and 75th percentiles as determined by R software; whiskers extend 1.5 times the interquartile range from the 25th and 75th percentiles; outliers are represented by dots. *n* = 5 sample points. Numbers above the boxes indicate the average competitive index (CI) in log_10_. *Significant difference relative to the wild‐type (WT) (*P* < 0.05). **Absence of any significant difference relative to WT (*P* > 0.05). Statistical analysis was performed with the Mann–Whitney *U*‐test.

Amino acid auxotrophic mutants (Cys^–^, Leu^–^, Met^–^ and Lys^–^) tested in co‐inoculation experiments could be phenotypically complemented *in planta*. The addition of both the non‐synthesized amino acid and the auxotrophic mutant to the wound totally or almost completely suppressed the growth defect of the auxotrophic mutant *in planta* (Fig. 5), confirming the low availability of certain amino acids in chicory. These results confirmed that Tn‐seq is a reliable technique to identify genes involved in plant colonization.

## Conclusion

This Tn‐seq experiment highlights some new factors required for the successful rotting of chicory by *D. dadantii*. Many genes known to be important for pathogenesis were not found in this screen because their products are secreted and can be shared with other strains in the community. This includes all the proteins secreted by the type II secretion system and small molecules, such as siderophores and butanediol. Other categories of genes, for example those involved in the response to acidic or oxidative stresses, were not found. Hence, chicory has been described as an inadequate model for the study of the response of *D. dadantii* to oxidative stress (Santos *et al.*, 2001). Similarly, the type III *hrp* genes were not identified in our study. The Hrp system is not always required for *D. dadantii* virulence and, in our experimental conditions (high inoculum on isolated chicory leaves), the necrotrophic capacities of *D. dadantii* (production of plant cell wall‐degrading enzymes) are probably sufficient on their own to provoke the disease. Our results also reveal some previously unknown aspects of the infection process. The struggle between plant and bacterial pathogens for iron supply has been well described. However, a competition for amino acids and nucleic acid also seems to occur in the plant. The level of nucleic acids and of the amino acids Cys, Leu, Met, Thr and Ile is too low in chicory to allow an efficient multiplication of bacteria defective in their biosynthesis. *Pectobacterium carotovorum* ssp. *carotovorum* Pcc21 appears to encounter almost the same conditions of nutrient deprivation when infecting Chinese cabbage (Hinton *et al.*, 1989; Lee *et al.*, 2013; Pirhonen *et al.*, 1991).

Some enzymatic steps involved in their synthesis are specific to bacteria and fungi. Thus, they could constitute good targets for the development of specific inhibitors (Thangavelu *et al.*, 2015) to prevent *D. dadantii *infections*. *The regulation of *D. dadantii *virulence has been studied extensively (Charkowski *et al.*, 2012; Reverchon *et al.*, 2016). However, new regulatory genes were also detected in this study. New members of the FlhDC regulation pathway were also detected. A few genes of unknown function remain to be studied.

*Dickeya dadantii* can infect dozens of plants. In addition to chicory, *D. dadantii* virulence tests are usually performed on potato plants, tubers or slices, *Arabidopsis thaliana*, Saintpaulia and celery. The metabolic status or reaction defences of these model plants are all different and the bacterial genes required for successful infection will probably differ in each model. Testing of several models would reveal the full virulence repertoire of the bacterium.

Although Tn‐seq has been used to study genes required for the infection of animals, there has been no genome‐wide study of the factors necessary for a necrotrophic plant pathogen to develop and provoke disease on a plant. In addition to the genes of known function described in the Results and discussion section, this study identified several genes of unknown function required for chicory rotting. Repetition of these experiments with other strains and on other plants will clarify whether these genes encode strain‐ or host‐specific virulence factors.

## Experimental Details

### Bacterial strains and growth conditions

The bacterial strains, phages, plasmids and oligonucleotides used in this study are described in Tables S3–S5 (see Supporting Information). *Dickeya dadantii* and *E. coli* cells were grown at 30 and 37 °C, respectively, in LB medium or M63 minimal medium supplemented with glycerol (2 g/L). When required, antibiotics were added at the following concentrations: ampicillin, 100 µg/L; kanamycin and chloramphenicol, 25 µg/L. Media were solidified with 1.5 g/L agar. Transduction with phage PhiEC2 was performed according to Résibois *et al.* (1984).

### Construction of the transposon library

Five mL of an overnight culture of *D. dadantii* strain A350 and of *E. coli* MFDpir/pSamEC were mixed and centrifuged for 2 min at 6000 *g*. The bacteria were resuspended in 1 mL of M63 medium and spread onto a 0.45‐µm cellulose acetate filter placed on a M63 medium agar plate. After 8 h, bacteria were resuspended in 1 mL of M63 medium. An aliquot was diluted and spread onto LB agar + kanamycin plates to estimate the efficiency of mutagenesis. The remaining culture was inoculated into 100 mL of LB medium + kanamycin and grown for 24 h at 30 °C. To confirm that the bacteria that grew were *D. dadantii* strains with a transposon, but without plasmid pSamEC, we checked that all the grown bacteria were kanamycin‐resistant (kan^R^), ampicillin‐susceptible (amp^S^) and diaminopimelate (DAP) prototrophs (MFDpir is DAP^–^). The bacteria were frozen in 40% glycerol at −80 °C and represent a library of about 300 000 mutants.

### DNA preparation for high‐throughput sequencing

An aliquot of the mutant library was grown overnight in LB medium + kanamycin. To identify the essential genes in LB, the culture was diluted 1000‐fold in LB medium and grown for 6 h. To infect chicory, the overnight culture was centrifuged and resuspended at an optical density at 600 nm (OD_600_) = 1 in M63 medium. Chicory plants, bought at a local grocery store, were cut in half, inoculated with 10 µL of this bacterial suspension and incubated at 30 °C with maximum moisture. After 60 h, rotten tissue was collected and filtered through cheesecloth. The bacteria were collected by centrifugation and washed twice in M63 medium. DNA was extracted from 1.5‐mL aliquots of bacterial suspension adjusted to OD_600_ = 1.5 with a Promega Wizard Genomic DNA Purification Kit (Promega, Madison, WI, USA). The subsequent steps of the DNA preparation methods were adapted from Skurnik *et al.* (2013). All DNA gel extractions were performed on a blue‐light transilluminator of DNA stained with GelGreen (Biotium, Fremont, CA, USA) to avoid DNA mutation and double‐strand breaks. Fifty micrograms of DNA sample were digested with 50 U MmeI in a total volume of 1.2 mL for 1 h at 37 °C according to the manufacturer’s instructions, heat inactivated for 20 min at 80 °C, purified (QIAquick PCR Purification Kit, Qiagen) and concentrated using a vacuum concentrator to a final volume of 25 µL. Digested DNA samples were run on a 1% agarose gel, the 1.0–1.5‐kb band containing the transposon and adjacent DNA was cut out and DNA was extracted from the gel according to the manufacturer’s instructions (QIAquick Gel Extraction Kit, Qiagen). This allowed the recovery of all the fragments containing genomic DNA adjacent to the transposons (1201 bp of transposable element with 32–34 bp of genomic DNA). A pair of single‐stranded complementary oligonucleotides containing a unique five‐nucleotide barcode sequence (LIB_AdaptT and LIB_AdaptB) was mixed and heated to 100 °C, and then slowly cooled down in a water bath to obtain double‐stranded adaptors with two‐nucleotide overhangs. One µg of DNA of each sample was ligated to the barcoded adaptors (0.44 mM) with 2000 U T4 DNA ligase in a final volume of 50 µL at 16 °C overnight. Five identical polymerase chain reactions (PCRs) from the ligation product were performed to amplify the transposon adjacent DNA. One reaction contained 100 ng of DNA, 1 U of Q5 DNA polymerase (Biolabs, Ipswich, MA, USA), 1 × Q5 buffer, 0.2 mM dNTPs, and 0.4 µm of the forward primer (LIB_PCR_5, which anneals to the P7 Illumina sequence of the transposon) and the reverse primer (LIB_PCR_3, which anneals to the P5 adaptor). Only 18 cycles were performed to keep a proportional amplification of the DNA. Samples were concentrated using a vacuum concentrator to a final volume of 25 µL. Amplified DNA was run on a 1.8% agarose gel, and the 125‐bp band was cut out and gel extracted (QIAquick PCR Purification Kit, Qiagen). DNA was finally dialysed (MF‐Millipore™ Membrane Filters) for 4 h. Quality control of the Tn‐seq DNA libraries (size of the fragments and concentration) and high‐throughput sequencing on HiSeq 2500 (Illumina, San Diego, CA, USA) were performed by MGX (CNRS Sequencing Service, Montpellier, France). After demultiplexing, the total number of reads was between 18 and 31 million (Table 1).

### Bioinformatics analysis

Differences in sequencing yields between samples were normalized by randomly subsampling each sample (i.e. rarefaction) to the lowest sequencing yield (the chicory #1 sample with 18 748 028 reads). Raw reads from the fastQ files were first filtered using cutadapt v1.11 (Martin, 2011) and only reads containing the *mariner* inverted left repeat (ACAGGTTGGATGATAAGTCCCCGGTCTT) were trimmed and considered as *bona fide* transposon‐disrupted genes. Trimmed reads were then analysed using a modified version of the TPP script available from TRANSIT software version 2.0.2 (Dejesus *et al.*, 2015). The mapping step was modified to select only those reads mapping uniquely and without mismatch in the *D. dadantii *3937 genome (GenBank CP002038.1). Then, the counting step was modified to accurately count the reads mapping to each TA site in the reference genome according to the Tn‐seq protocol used in this study. Read counts per insertion were normalized using the LOESS method, as described in Zomer *et al.* (2012). Finally, TRANSIT software (version 2.0) was used to compare the Tn‐seq datasets.

### Strain construction

To construct the A4277 strain, gene Dda3937_03424 was amplified with the oligonucleotides 19732+ and 19732–. The resulting fragment was inserted into the pGEM‐T plasmid (Promega). A *uidA*‐kan^R^ cassette (Bardonnet and Blanco, 1991) was inserted into the unique *Age*I site of the fragment. The construct was recombined into the *D. dadantii* chromosome according to Roeder and Collmer (1985). Recombination was checked by PCR. To construct the in‐frame deletion mutants, the counter‐selection method using the *sacB* gene was employed (Link *et al.*, 1997). The suicide pRE112 plasmid containing 500 bp of upstream and downstream DNA of the gene to be deleted was transferred by conjugation from the *E. coli* *MFDpir* strain into *D. dadantii *3937. Selection of the first event of recombination was performed on LB agar supplemented with chloramphenicol at 30 µg/L. Transconjugants were then spread on LB agar without NaCl and supplemented with 5% sucrose to allow the second event of recombination. In‐frame deletions were checked by auxotrophy analysis and/or by PCR (Dreamtaq polymerase, Thermofisher, Waltham, MA, USA). In order to discriminate mutants from the WT strain during co‐inoculation experiments, a Gentamicine‐resistant (Gm^R^) derivative of the WT strain was constructed by insertion of the mini‐Tn*7*‐Gm into the *att*Tn7 site (close to the *glmS* gene) (Zobel *et al.*, 2015). A 3937 Gm^R^ strain was made by co‐electroporation of pTn7‐M (Zobel *et al.*, 2015) and pTnS3 (Choi *et al.*, 2008) plasmids into the *D. dadantii* 3937 strain. The mini‐Tn*7*‐Gm delivered by the pTn7‐M vector (suicide plasmid in *D. dadantii*) was inserted into the *att*Tn7 site (close to the *glmS* gene) of the recipient strain thanks to the pTnS3 plasmid encoding the Tn*7* site‐specific transposition pathway. The Gm^R^ strain obtained was then checked by PCR using attTn7‐Dickeya3937‐verif and 3‐Tn7L primers (Table S5).

### Protein techniques

Flagella were prepared from cells grown overnight in LB medium. Bacteria were pelleted, resuspended in 1/10 volume of water and passed 20‐fold through a needle on a syringe. Cells and cell debris were removed by centrifugation for 5 min at 20 000 *g* (Shevchik *et al.*, 1994). Proteins were analysed by SDS‐PAGE. The molecular mass of flagellin was determined by matrix‐assisted laser desorption/ionization‐mass spectrometry (MALDI‐MS) at the Biopark Platform at Archamps, France.

### Celery inoculation experiments

Celery plants were bought at a local grocery store. The WT and A4277 (glycosylation) mutant were grown overnight in M63 + glycerol medium. Bacteria were washed in M63 medium and OD_600_ was adjusted to 1.0. Bacteria were diluted 10‐fold in the same medium. Ten microlitres of the bacterial suspension were inoculated into a hole in the leaves that had been made with a pipette tip. The wound was covered with mineral oil and the leaves were incubated at 30 °C at high humidity for 2 days. The length of rotten tissue was measured.

### Co‐inoculation experiments

To determine the CI of the mutants, the WT strain and the test mutant were grown overnight in LB medium. Bacteria were washed in M63 medium and the OD_600_ was adjusted to 1.0. Bacteria were mixed in a 1 : 1 ratio and diluted 10‐fold. For complementation experiments *in planta*, the dilution was performed in M63 medium with 1 mm of the required amino acid. Ten microlitres of the mixture were inoculated into chicory leaves. The wound was covered with mineral oil and the leaves were incubated at 30 °C at high humidity. After 24 h, rotten tissue was collected, homogenized, diluted in M63 and spread onto LB and LB + antibiotic plates. After 48 h at 30 °C, the colonies were counted. The CI is the ratio: (number of mutant bacteria/number of WT bacteria) in rotten tissue/(number of mutant bacteria/number of WT bacteria) in the inoculum. For the genes whose absence confers a growth advantage in chicory according to the Tn‐seq experiment, in‐frame deletions were realized in a WT strain. The other mutants were constructed in the 3937 Gm^R^ strain. This allows an easy detection of clones of the under‐represented strain among those of the other strain.

### Nucleotide sequence accession numbers

The transposon sequence reads obtained have been submitted to the European Nucleotide Archive (ENA) database under accession number PRJEB20574.

## Supporting information


**Fig. S1** Volcano plot of RESAMPLING results comparing replicates grown in chicory versus in Luria–Bertani (LB) medium. Significant hits have *q* < 0.05 or −log_10_ *q* > 1.3. Growth defect (GD) and growth advantage (GA) genes are indicated by a red frame.

**Fig. S2** Examples of essential and important genes revealed by transposon sequencing (Tn‐seq). Number of reads at each transposon location in the sample grown in either Luria–Bertani (LB) medium or chicory. Data are averaged from biological replicates and normalized as described in Experimental details. Four regions of the genome representative of the Tn‐seq results are shown, with the predicted genes indicated at the bottom of each panel. Peaks represent the read number at TA sites. Black arrows represent genes that passed the permutation test (*q*‐value ≤ 0.05). Small arrows indicate the presence of a promoter. (A) *dnaX*, which encodes both the τ and γ subunits of DNA polymerase, is represented by a grey arrow. *dnaX* is an essential gene in LB. *acrAB* genes represented by dark arrows are growth defect (GD) genes in chicory (*q*‐value ≤ 0.05). (B) Essentiality of leucine biosynthetic genes in chicory. (C) Importance of genes involved in motility for growth in chicory. (C) Insertions in the 5′ region of *rsmC* confer a growth advantage for the bacterium in chicory.

**Table S1** Raw data of the Hidden Markov Model (HMM) and resampling analysis by TRANSIT.

**Table S2** Number of genes implicated in the Kyoto Encyclopedia of Genes and Genomes (KEGG) pathway.

**Table S3** Bacterial strains used in this study.

**Table S4** Plasmids used in this study.

**Table S5** Oligonucleotides used in this study.
