# Supplementary figures and images for: Identification by Tn‐seq of Dickeya dadantii genes required for survival in chicory plants
Source: Mol Plant Pathol. 2018 Nov 15;20(2):287–306. doi: 10.1111/mpp.12754 (PMC6637903; doi:10.1111/mpp.12754)

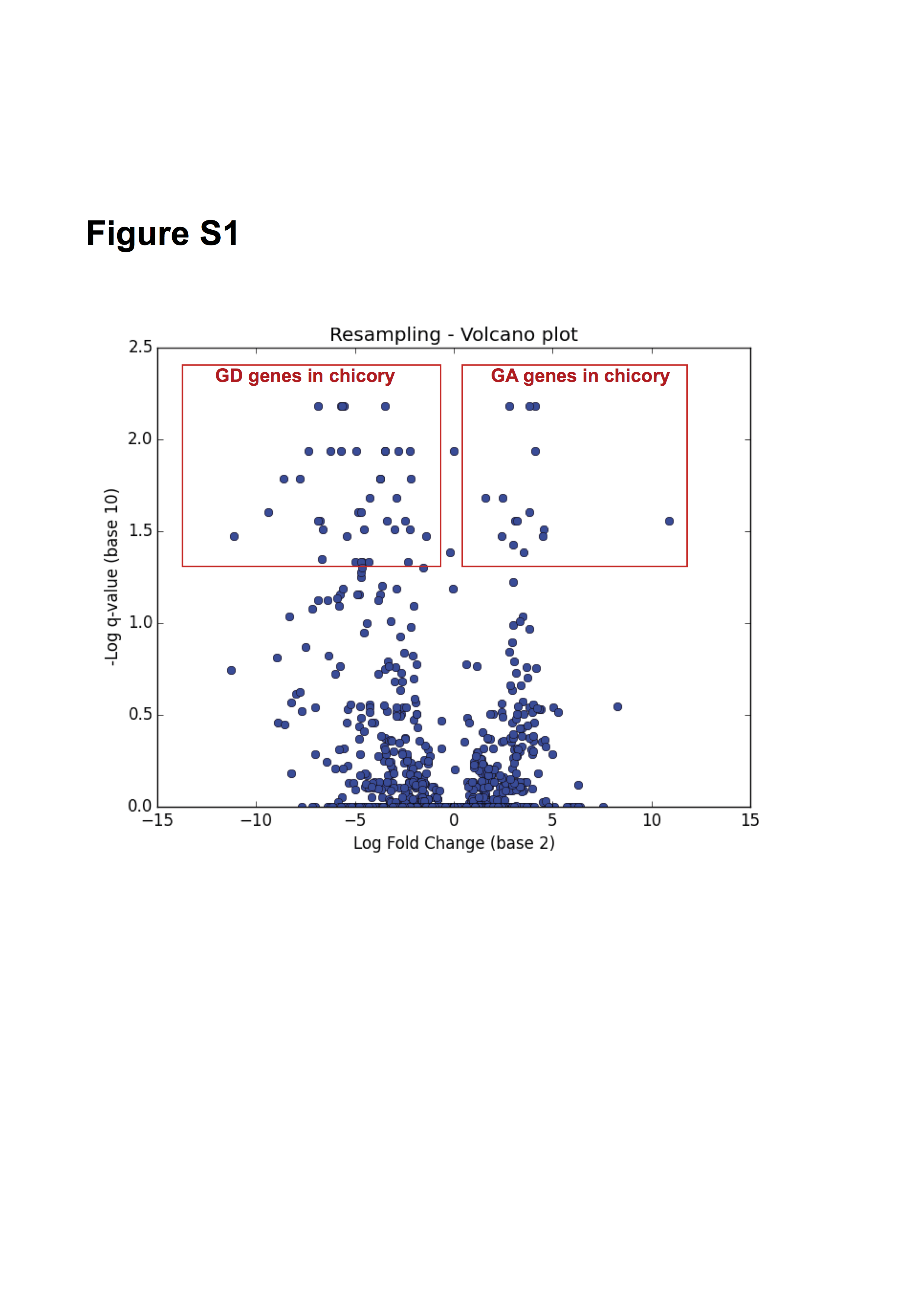

Supplement: Supplementary file 1 — Fig. S1 Volcano plot of RESAMPLING results comparing replicates grown in chicory versus in Luria–Bertani (LB) medium. Significant hits have q < 0.05 or −log10 q > 1.3. Growth defect (GD) and growth advantage (GA) genes are indicated by a red frame. [file MPP-20-287-s001.tiff]

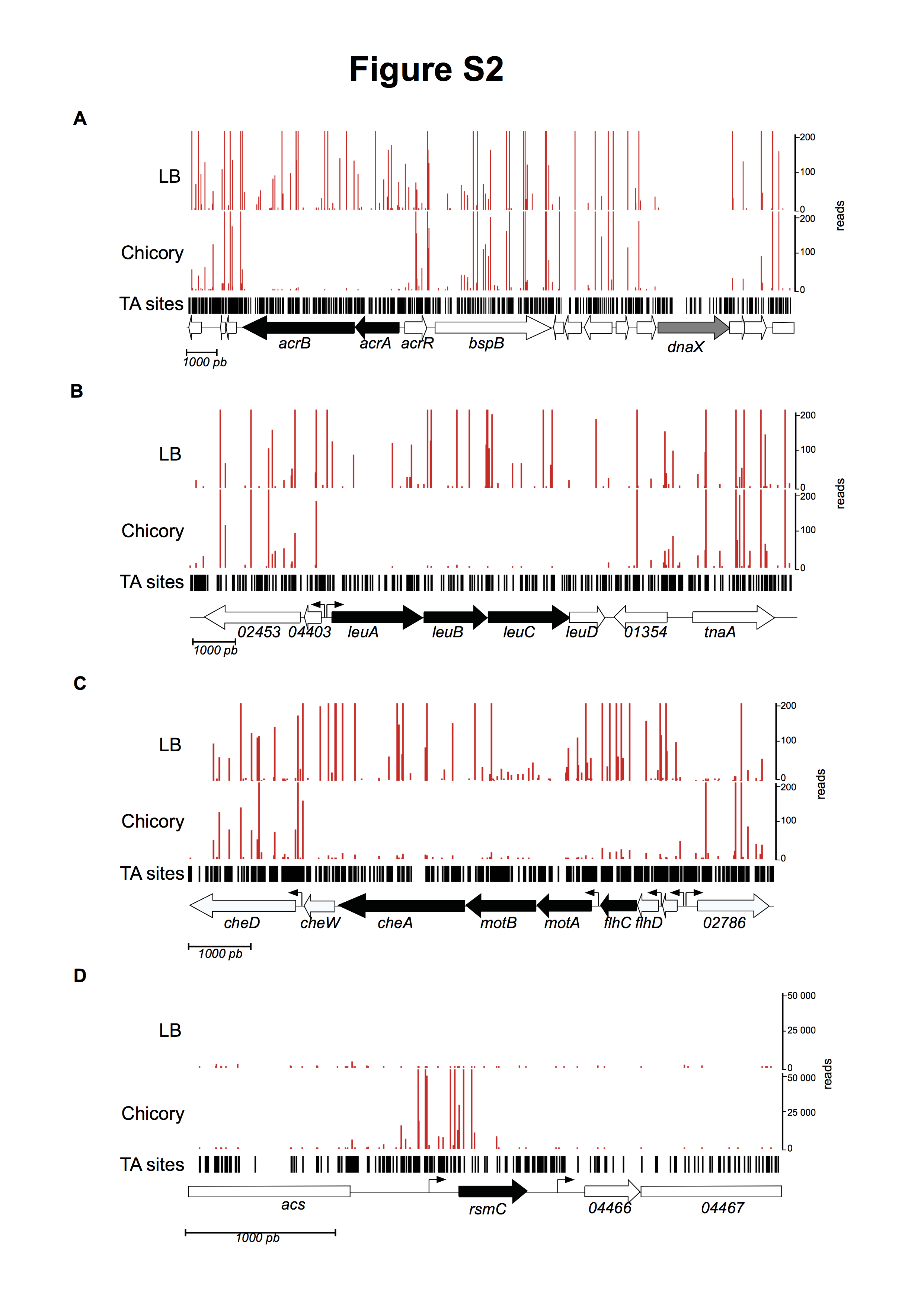

Supplement: Supplementary file 2 — Fig. S2 Examples of essential and important genes revealed by transposon sequencing (Tn‐seq). Number of reads at each transposon location in the sample grown in either Luria–Bertani (LB) medium or chicory. Data are averaged from biological replicates and normalized as described in Experimental details. Four regions of the genome representative of the Tn‐seq results are shown, with the predicted genes indicated at the bottom of each panel. Peaks represent the read number at TA sites. Black arrows represent genes that passed the permutation test (q‐value ≤ 0.05). Small arrows indicate the presence of a promoter. (A) dnaX, which encodes both the τ and γ subunits of DNA polymerase, is represented by a grey arrow. dnaX is an essential gene in LB. acrAB genes represented by dark arrows are growth defect (GD) genes in chicory (q‐value ≤ 0.05). (B) Essentiality of leucine biosynthetic genes in chicory. (C) Importance of genes involved in motility for growth in chicory. (C) Insertions in the 5′ region of rsmC confer a growth advantage for the bacterium in chicory. [file MPP-20-287-s002.tif]
