# Supplementary material for: Identification by Tn‐seq of Dickeya dadantii genes required for survival in chicory plants
Source: Mol Plant Pathol. 2018 Nov 15;20(2):287–306. doi: 10.1111/mpp.12754 (PMC6637903; doi:10.1111/mpp.12754)
Supplement: Supplementary file 4 — Table S2 Number of genes implicated in the Kyoto Encyclopedia of Genes and Genomes (KEGG) pathway. [file MPP-20-287-s004.docx]

**TABLE S2** Number of genes implicated in KEGG pathway

| **KEGG pathway**  **No.** | **Pathway** | **No. of genes**  **implicated** |
| --- | --- | --- |
| ddd01100 | Metabolic pathways | 30 |
| ddd02040 | Flagellar assembly | 19 |
| ddd01110 | Biosynthesis of secondary metabolites | 15 |
| ddd00230 | Purine metabolism | 8 |
| ddd02020 | Two-component system | 8 |
| ddd01120 | Microbial metabolism in diverse environments | 8 |
| ddd00240 | Pyrimidine metabolism | 7 |
| ddd02030 | Bacterial chemotaxis | 6 |
| ddd00920 | Sulfur metabolism | 4 |
| ddd00250 | Alanine, aspartate and glutamate metabolism | 4 |
| ddd01200 | Carbon metabolism | 4 |
| ddd00680 | Methane metabolism | 3 |
| ddd01210 | 2-Oxocarboxylic acid metabolism | 3 |
| ddd00620 | Pyruvate metabolism | 3 |
| ddd00290 | Valine, leucine and isoleucine biosynthesis | 3 |
| ddd01503 | Cationic antimicrobial peptide (CAMP) resistance | 3 |
| ddd00670 | One carbon pool by folate | 2 |
| ddd02060 | Phosphotransferase system (PTS) | 2 |
| ddd00430 | Taurine and hypotaurine metabolism | 2 |
| ddd00450 | Selenocompound metabolism | 2 |
| ddd00640 | Propanoate metabolism | 2 |
| ddd00660 | C5-Branched dibasic acid metabolism | 2 |
| ddd00030 | Pentose phosphate pathway | 2 |
| ddd00010 | Glycolysis / Gluconeogenesis | 2 |
| ddd00750 | Vitamin B6 metabolism | 2 |
| ddd02010 | ABC transporters | 2 |
