# Supplementary material for: Identification by Tn‐seq of Dickeya dadantii genes required for survival in chicory plants
Source: Mol Plant Pathol. 2018 Nov 15;20(2):287–306. doi: 10.1111/mpp.12754 (PMC6637903; doi:10.1111/mpp.12754)
Supplement: Supplementary file 5 — Table S3 Bacterial strains used in this study. [file MPP-20-287-s005.docx]

Table S3: bacterial strains used in this study

| **Strain** | **Description** | **Reference** |
| --- | --- | --- |
| *Escherichia coli* K12 | |  |
| MFD*pir* | *RP4-2-Tc::(∆Mu1::aac(3)IV-∆aphA-·∆nic35-∆Mu2::zeo) ∆dapA::erm-pir) ∆recA* | [[1]](https://paperpile.com/c/Lkyhht/mE2i) |
| DH5αλpir | λpir phage lysogen of DH5α | Laboratory collection |
|  |  |  |
| *Dickeya dadantii* | |  |
| 3937 | Wild type strain | Laboratory collection |
| A3422 | *Dda3937_03419*::Mu-kan^R^ | Laboratory collection |
| A4277 | *Dda3937_03424*::*uidA*-kan^R^ | Laboratory collection |
| D7#1 | *D. dadantii 3937 glmS::Tn7-gent*, Gent^R^ | This study |
| D8 | 3937 ∆*rsmC* | This study |
| D10 | D7#1 ∆*degQ,* Gent^R^ | This study |
| D15 | 3937 ∆*gcpA* | This study |
| D17 | D7#1 ∆*metB,* Gent^R^ | This study |
| D19 | D7#1 ∆*lysA,* Gent^R^ | This study |
| D21 | D7#1 ∆*purF,* Gent^R^ | This study |
| D23 | D7#1 ∆*pyrE,* Gent^R^ | This study |
| D25 | D7#1 ∆*clpSA,* Gent^R^ | This study |
| D27 | D7#1 ∆*guaB,* Gent^R^ | This study |
| D29 | D7#1 ∆*leuA,* Gent^R^ | This study |
| D31 | D7#1 ∆*purL,* Gent^R^ | This study |
| D33 | D7#1 ∆*cysJ,* Gent^R^ | This study |
| D35 | D7#1 ∆*hdfR,* Gent^R^ | This study |
| D37 | D7#1 ∆*flhDC,* Gent^R^ | This study |
| D39 | D7#1 ∆*carA,* Gent^R^ | This study |

1. Ferrières L, Hémery G, Nham T, Guérout A-M, Mazel D, Beloin C, et al. Silent mischief: bacteriophage Mu insertions contaminate products of Escherichia coli random mutagenesis performed using suicidal transposon delivery plasmids mobilized by broad-host-range RP4 conjugative machinery. J Bacteriol. 2010;192: 6418–6427.
