# Supplementary material for: Identification by Tn‐seq of Dickeya dadantii genes required for survival in chicory plants
Source: Mol Plant Pathol. 2018 Nov 15;20(2):287–306. doi: 10.1111/mpp.12754 (PMC6637903; doi:10.1111/mpp.12754)
Supplement: Supplementary file 6 — Table S4 Plasmids used in this study. [file MPP-20-287-s006.docx]

Table S4: plasmids used in this study

| **Plasmid** | **Description** | **Reference** |
| --- | --- | --- |
| pSAM-Ec | Suicide mobilizable vector; Amp^R^, Km^R^ resistance gene bordered by mariner inverted repeat sequence containing MmeI restriction site, himar1-C9 transposase gene under the control of P*lac.* | [[1]](https://paperpile.com/c/Lkyhht/Nnga) |
| pRE112 | Suicide vector for allelic exchange in *D. dadantii* 3937, Cm^R^ ,*sacB*, *oriT* RP4 | [[2]](https://paperpile.com/c/Lkyhht/Nnga) |
| pTn7-M | Km^R^ Gm^R^, *ori R6K*,*Tn7L* and *Tn7R* extremities, standard multiple cloning site, *oriT* RP4 | [[3]](https://paperpile.com/c/Lkyhht/Nnga) |
| pTNS3 | Ap^R^, *ori R6K*,*TnsABCD* operon, *oriT* RP4 | [4] |
| pRE112 *ΔcysJ* | *Dickeya dadantii* 3937, *cysJ* deletion plasmid, Cm^R^ | This study |
| pRE112 *ΔgcpA* | *Dickeya dadantii* 3937, *gcpA* deletion plasmid, Cm^R^ | This study |
| pRE112 *ΔdegQ* | *Dickeya dadantii* 3937, *degQ* deletion plasmid, Cm^R^ | This study |
| pRE112 *ΔflhDC* | *Dickeya dadantii* 3937, *flhDC* deletion plasmid, Cm^R^ | This study |
| pRE112 *ΔguaB* | *Dickeya dadantii* 3937, *guaB* deletion plasmid, Cm^R^ | This study |
| pRE112 *ΔmetB* | *Dickeya dadantii* 3937, *metB* deletion plasmid, Cm^R^ | This study |
| pRE112 *ΔlysA* | *Dickeya dadantii* 3937, *lysA* deletion plasmid, Cm^R^ | This study |
| pRE112 *ΔpyrE* | *Dickeya dadantii* 3937, *pyrE* deletion plasmid, Cm^R^ | This study |
| pRE112 *ΔpurL* | *Dickeya dadantii* 3937, *purL* deletion plasmid, Cm^R^ | This study |
| pRE112 *ΔpurF* | *Dickeya dadantii* 3937, *purF* deletion plasmid, Cm^R^ | This study |
| pRE112 *ΔcarA* | *Dickeya dadantii* 3937, *carA* deletion plasmid, Cm^R^ | This study |
| pRE112 *ΔclpS/A* | *Dickeya dadantii* 3937, *clpS/A* deletion plasmid, Cm^R^ | This study |
| pRE112 *ΔhdfR* | *Dickeya dadantii* 3937, *hdfR* deletion plasmid, Cm^R^ | This study |
| pRE112 *ΔleuA* | *Dickeya dadantii* 3937, *leuA* deletion plasmid, Cm^R^ | This study |
| pRE112 *ΔrsmC* | *Dickeya dadantii* 3937, *rsmC* deletion plasmid, Cm^R^ | This study |

1. Wiles TJ, Norton JP, Russell CW, Dalley BK, Fischer KF, Mulvey MA. Combining quantitative genetic footprinting and trait enrichment analysis to identify fitness determinants of a bacterial pathogen. PLoS Genet. 2013;9: e1003716.
2. Edwards RA, Keller LH, Schifferli DM. Improved allelic exchange vectors and their use to analyze 987P fimbria gene expression. Gene. 1998 Jan 30;207(2):149-57.
3. Zobel S, Benedetti I, Eisenbach L, de Lorenzo V, Wierckx N, Blank LM. Tn7-Based Device for Calibrated Heterologous Gene Expression in Pseudomonas putida. ACS Synth Biol. 2015 Dec 18;4(12):1341-51.
4. Choi KH, Mima T, Casart Y, Rholl D, Kumar A, Beacham IR, Schweizer HP. Genetic tools for select-agent-compliant manipulation of Burkholderia pseudomallei. Appl Environ Microbiol. 2008 Feb;74(4):1064-75
