# Supplementary material for: Identification by Tn‐seq of Dickeya dadantii genes required for survival in chicory plants
Source: Mol Plant Pathol. 2018 Nov 15;20(2):287–306. doi: 10.1111/mpp.12754 (PMC6637903; doi:10.1111/mpp.12754)
Supplement: Supplementary file 7 — Table S5 Oligonucleotides used in this study. [file MPP-20-287-s007.docx]

Table S5: oligonucleotides used in this study

| **Oligonucleotide** | **Sequence^a^** | **Reference** |
| --- | --- | --- |
| 19732+ | GTGACCATGCCAGATACACAACC | This study |
| 19732- | TCACAACAGGTTCTTTAACGCATC | This study |
| LIB_AdaptT | TTCCCTACACGACGCTCTTCCGATCTNN | [[5]](https://paperpile.com/c/Lkyhht/cOVQ) |
| LIB_AdaptB | AGATCGGAAGAGCGTCGTGTAGGGAA | [[5]](https://paperpile.com/c/Lkyhht/cOVQ) |
| LIB_PCR_5 | CAAGCAGAAGACGGCATACGAAGACCGGGGACTTATCATCCAACCTGT | [[5]](https://paperpile.com/c/Lkyhht/cOVQ) |
| LIB_PCR_3 | AATGATACGGCGACCACCGAACACTCTTTCCCTACACGACGCTCTTCCGATCT | [[5]](https://paperpile.com/c/Lkyhht/cOVQ) |
| rsmC-sacI-up | *CATGAATTCCCGGGA****GAGCTC***GGACGATATGATCAAACAAG | This study |
| rsmC-start-rev | TGACGCCATCAGACTCATATGTCCTCTGCTTC | This study |
| rsmC-stop-fwd | ATGAGTCTGATGGCGTCATAAAACCGGATG | This study |
| KpnI-rsmC | *CAAGCTTCTTCTAGA****GGTACC***ATCGCTCAGGGACACGACC | This study |
| pyrE-SacI-up | *CATGAATTCCCGGGA****GAGCTC***ATTGGATTGCGATGTGTTG | This study |
| pyrE-start-rev | TTACACGCCGAAATAGGCTTTCATGACTCCCTC | This study |
| pyrE-stop-fwd | ATGAAAGCCTATTTCGGCGTGTAAAACAGTTC | This study |
| pyrE-KpnI-dwn | *CAAGCTTCTTCTAGA****GGTACC***TGACAGCCTGATCGAATTTATTG | This study |
| purL-SacI-up | *CATGAATTCCCGGGA****GAGCTC***GAATCGTTTGGCCAGTTCG | This study |
| purL-start-rev | CCAACTGACTATTTCCATCATCTAAATTTCTCTCG | This study |
| purL-stop-fwd | TGATGGAAATAGTCAGTTGGGGTAATGCGATAAATTAATG | This study |
| purL-KpnI-dwn | *CAAGCTTCTTCTAGA****GGTACC***TCGCCCGATGTGTGATAAAG | This study |
| purF-sacI-up | *CATGAATTCCCGGGA****GAGCTC***ATTGGATTGCGATGTGTTGC | This study |
| purF-start-rev | CACGCCGAACTGGGCTTTCATGACTCCCTCTC | This study |
| purF-stop-fwd | ATGAAAGCCCAGTTCGGCGTGTAAAACAG | This study |
| purF-KpnI-dwn | *CAAGCTTCTTCTAGA****GGTACC***CGCATCAACCTGATTCTG | This study |
| carA-SacI-up | *CATGAATTCCCGGGA****GAGCTC***GCTTGATGATATATAAACGGGAAG | This study |
| carA-start-rev | ATTATTTGGTGTTTGACTTAATCAAAACACCCTCC | This study |
| carA-stop-fwd | GATTAAGTCAAACACCAAATAATCAGAATCCG | This study |
| carA-KpnI-dwn | *CAAGCTTCTTCTAGA****GGTACC***GATCTTCCGCTTTATCGATGG | This study |
| clpS-SacI-up | *CATGAATTCCCGGGA****GAGCTC***TGGTGCACGGTGAATTG | This study |
| clpS-start-rev | TCAGTGAACACTATGATTTCCCATCTTCTATCC | This study |
| clpA-stop-fwd | GGAAATCATAGTGTTCACTGAGTGAATTGAATACG | This study |
| clpA-KpnI-dwn | *CAAGCTTCTTCTAGA****GGTACC***GAGGATTCGATGGCCAAAG | This study |
| hdfR-SacI-up | *CATGAATTCCCGGGA****GAGCTC***ATGAACCCAATCTTGCATCA | This study |
| hdfR-start-rev | GCTATGTTCAGTTTCCTCCAAAAAATTTTATCGTCATG | This study |
| hdfR-stop-fwd | TTGGAGGAACTGAACATAGCTTCTTAAACCAC | This study |
| hdfR-KpnI-dwn | *CAAGCTTCTTCTAGA****GGTACC***AATCGGTGGTGCGGTAGTTC | This study |
| cysJ-SacI-up | *CATGAATTCCCGGGA****GAGCTC***TCAAAGCGGGCATGTTGATA | This study |
| cysJ-start-rev | CTCTCTGATACGAAGTTGTCATTGTGGTATCG | This study |
| cysJ-stop-fwd | ATGACAACTTCGTATCAGAGAGATGTGTACTAATGAGC | This study |
| cysJ-KpnI-dwn | *CAAGCTTCTTCTAGA****GGTACC***ACTCCACCGGGTTAGAGGTG | This study |
| gcpA-SacI-up | *CATGAATTCCCGGGA****GAGCTC***AGCAATCATAAGAGAAAGAACTG | This study |
| gcpA-start-rev | GTTTACTGGTGATCGAACAAATCGCATCACTG | This study |
| gcpA-stop-fwd | TTGTTCGATCACCAGTAAACGCCGATTCG | This study |
| gcpA-KpnI-dwn | *CAAGCTTCTTCTAGA****GGTACC***GACGCCAGTGAAAACAAG | This study |
| degQ-SacI-up | *CATGAATTCCCGGGA****GAGCTC***TCAGGAACTGGTCAGCCACT | This study |
| degQ-start-rev | CAACAGATACATTGATTGTTAACTCTCTCACGA | This study |
| degQ-stop-fwd | GTTAACAATCAATGTATCTGTTGCTGCGTTAAGC | This study |
| degQ-KpnI-dwn | *CAAGCTTCTTCTAGA****GGTACC***AGATCGGTCAGGCTGTCT | This study |
| flhDC-SacI-up | *CATGAATTCCCGGGA****GAGCTC***TGGTTGTAGAGTCGCGGTTT | This study |
| flhDC-start-rev | ACTGCATGAGA***GGTACC***CATATTCCCATC | This study |
| flhC-stop-fwd | ATG***GGTACC***TCTCATGCAGTCTGAGCCTGATG | This study |
| flhDC-KpnI-dwn | *CAAGCTTCTTCTAGA****GGTACC***AGAAAGGATGCGGGGATAA | This study |
| guaB-SacI-up | *CATGAATTCCCGGGA****GAGCTC***ATGCTTTCGGCCAGCAACT | This study |
| guaB-start-rev | AGCCCATACACGTAACATGGCAATATCTCACC | This study |
| guaB-stop-fwd | CCATGTTACGTGTATGGGCTAAGCGCGTTAC | This study |
| guaB-KpnI-dwn | *CAAGCTTCTTCTAGA****GGTACC***GTGGCTCATCCACACAT | This study |
| metB-Sac1-up | *CATGAATTCCCGGGA****GAGCTC***TTCCCACGTATCCGGGTC | This study |
| metB-start-rev | TTACCTCTTGGCTTTACGCGTCATCGGTGC | This study |
| KpnI-metB | *CAAGCTTCTTCTAGA****GGTACC***AGGAAGGTACGGGCATCC | This study |
| metB-stop-fwd | GACGCGTAAAGCCAAGAGGTAACAATGAGTG | This study |
| lysA-Sac1-up | *CATGAATTCCCGGGA****GAGCTC***AGATTGAGCGCCGGGTAAC | This study |
| lysA-start-rev | CACCCGCTCGTGTGGCATGGATATCGTACC | This study |
| KpnI-lysA | *CAAGCTTCTTCTAGA***GGTACC**GATCGATTATCTGGTGCAGC | This study |
| lysA-stop-fwd | ATGCCACACGAGCGGGTGTAACCGCTC | This study |
| leuA-Sac1-up | *CATGAATTCCCGGGA****GAGCTC***GAGCAATCTGCTGCTGAATG | This study |
| leuA-start-rev | TCACACTGTTTGGTTCATCGGTCTGGTTCC | This study |
| KpnI-leuA | *CAAGCTTCTTCTAGA****GGTACC***TCGAAACGGTGATACACCTC | This study |
| leuA-stop-fwd | ATGAACCAAACAGTGTGATGACCAAGAGTTAC | This study |
| attTn7-3937-verif | CGAATAGGGTTTCACGACAAAG | This study |
| 3-Tn7L | ATTAGCTTACGACGCTACACCC | This study |

^a^ restriction sites are underlined and in bold ; homology region with the pRE112 plasmid are in italic

5. Skurnik D, Roux D, Aschard H, Cattoir V, Yoder-Himes D, Lory S, et al. A comprehensive analysis of in vitro and in vivo genetic fitness of Pseudomonas aeruginosa using high-throughput sequencing of transposon libraries. PLoS Pathog. 2013;9: e1003582.
